# CAB-Align: A Flexible Protein Structure Alignment Method Based on the Residue-Residue Contact Area

**DOI:** 10.1371/journal.pone.0141440

**Published:** 2015-10-26

**Authors:** Genki Terashi, Mayuko Takeda-Shitaka

**Affiliations:** School of Pharmacy, Kitasato University, Tokyo, Japan; UMR-S665, INSERM, Université Paris Diderot, INTS, FRANCE

## Abstract

Proteins are flexible, and this flexibility has an essential functional role. Flexibility can be observed in loop regions, rearrangements between secondary structure elements, and conformational changes between entire domains. However, most protein structure alignment methods treat protein structures as rigid bodies. Thus, these methods fail to identify the equivalences of residue pairs in regions with flexibility. In this study, we considered that the evolutionary relationship between proteins corresponds directly to the residue–residue physical contacts rather than the three-dimensional (3D) coordinates of proteins. Thus, we developed a new protein structure alignment method, contact area-based alignment (CAB-align), which uses the residue–residue contact area to identify regions of similarity. The main purpose of CAB-align is to identify homologous relationships at the residue level between related protein structures. The CAB-align procedure comprises two main steps: First, a rigid-body alignment method based on local and global 3D structure superposition is employed to generate a sufficient number of initial alignments. Then, iterative dynamic programming is executed to find the optimal alignment. We evaluated the performance and advantages of CAB-align based on four main points: (1) agreement with the gold standard alignment, (2) alignment quality based on an evolutionary relationship without 3D coordinate superposition, (3) consistency of the multiple alignments, and (4) classification agreement with the gold standard classification. Comparisons of CAB-align with other state-of-the-art protein structure alignment methods (TM-align, FATCAT, and DaliLite) using our benchmark dataset showed that CAB-align performed robustly in obtaining high-quality alignments and generating consistent multiple alignments with high coverage and accuracy rates, and it performed extremely well when discriminating between homologous and nonhomologous pairs of proteins in both single and multi-domain comparisons. The CAB-align software is freely available to academic users as stand-alone software at http://www.pharm.kitasato-u.ac.jp/bmd/bmd/Publications.html.

## Introduction

During evolution, protein structures are more highly conserved than nucleotide sequences [1,2]. Therefore, comparing protein structures is a fundamental approach for homology detection, classification, and functional annotation for novel protein structures [3–5]. In particular, structural alignment, which assigns amino acids that are equivalent among proteins, is very useful for detecting functional sites and conserved positions. Many protein structure alignment methods have been developed in the past two decades. Most of these alignment methods aim to find the best alignment yielding the maximum number of equivalent amino acids between proteins with minimal structural deviation following three-dimensional (3D) coordinate superposition. These methods treat protein structures as rigid bodies and are categorized as “rigid-body alignment” approaches; these include DALI, FAST, CE, MAMMOTH, TM-align, and Fr-TM-align [6–11]. The superposition of protein structures in Cartesian space, such as RMS fitting, is sufficiently fast and accurate [12]. Thus, rigid-body alignment is widely used to find remote homologs when only proteins with low shared sequence identities are available.

However, proteins are known to be flexible, and this flexibility has an essential role in their functions, such as catalysis, protein–ligand interactions, and protein–protein interactions [13–14]. Rigid-body alignment methods cannot find correct alignments for proteins that undergo structural changes (almost all proteins exhibit small/large movements), and thus they fail to identify structural similarities in flexible regions. To overcome these issues, a flexible protein structure alignment approach has been developed called FATCAT [15], which finds the optimal structure alignment with the least number of rigid body movements using a dynamic programming (DP) algorithm to connect aligned fragment pairs (AFPs). In the DP algorithm, FATCAT uses the score calculated from the RMSD of AFPs and the number of twists. Therefore, FATCAT depends on the 3D coordinate superposition of AFPs. In addition, a flexible and translation/rotation-invariant alignment method has been proposed that does not use 3D coordinate superposition, which is called maximum contact map overlap (CMO) [16]. In CMO, the protein structure is represented by residue–residue contact maps, which are defined by the Euclidean distance between the representative coordinates in the corresponding amino acids. The CMO algorithm has been studied widely. In particular, Andonov et al. [17] proposed an exact CMO algorithm using integer programming and Lagrangian relaxation. Wohlers et al. [18] proposed an approximated CMO score and reported its protein structure classification performance. Moreover, GR-Align [19] is a fast CMO heuristic method based on generalized graphlets and the graphlet degree-to-order graph. GR-Align is at least 79 times faster than TM-align according to the reported results.

Recently, it was shown that residue–residue contacts have strong relationships with correlated mutations in multiple sequence alignments [20]. It was also observed that residue–residue contacts guide protein folding, and they are highly informative for fold recognition [21–24]. Therefore, we considered that the evolutionary relationships between proteins should reflect residue–residue physical contacts directly, rather than 3D coordinates. Thus, the assembled residue–residue physical contacts should reflect a protein fold represented in terms of 3D coordinates.

Based on the same concept, Olechnovic et al. introduced a contact area difference (CAD)-based score for evaluating the structural similarity between a protein model and the native structure [25], where they proposed the use of the residue–residue contact area as a residue–residue physical contact. Thus, the CAD-score is an extended algorithm based on CMO methods. They showed that the CAD-score is a more robust evaluation score than the global distance test total score [26], which is based on 3D coordinate superposition. The CAD-score was also shown to be robust when assessing the accuracy of protein models for multidomain and protein–protein complexes, as well as single-domain proteins. The CAD-score is essentially unaffected by the domain arrangement, and it can be applied to the flexible alignment method.

In this study, we developed a new protein structure alignment method called contact area-based alignment (CAB-align), which uses the similarity of the residue–residue contact area. The main aim of CAB-align is to identify homologous relationships at the residue level between related protein structures. CAB-align comprises the following three main steps. First, CAB-align employs a rigid-body alignment method based on local structural similarity. Second, structural alignment is performed based on global 3D structure superposition to generate a sufficient number of initial alignments. Finally, a heuristic method (iterative DP) is executed based on the modified CAD-score to obtain the optimal alignment.

In various benchmarks for protein structure alignment, the alignment quality (AQ) is defined based on comparisons with manually curated gold standard alignments or using geometrical similarity measures. In general, gold standard alignments are used, such as in SISYPHUS [27], the Conserved Domain Database [28], and the Homologous Structure Alignment Database [29]. In these databases, experts consider both the geometric superposition and sequence-based alignment, as well as manually resolving any conflicts between them. There are also many types of geometric similarity measures, such as the similarity index, structural alignment score, match index [30], and template modeling score [31]. These measures achieve a balance between the alignment coverage and geometrical deviation after optimal superposition. Therefore, the superposition of 3D coordinates remains an essential component of structure alignment methods and AQ evaluations.

However, as mentioned above, our CAB-align method allows flexible protein structure alignments where proteins are flexible. Therefore, we could not use geometric similarities that employ superposition based on Cartesian coordinates to assess the performance of this alignment method, we employed four main evaluation approaches: (1) agreement with the gold standard alignment, (2) AQ based on an evolutionary relationship without the superposition of 3D coordinates, (3) consistency among multiple alignments, and (4) agreement with the gold standard classification.

We compared the performance of CAB-align with the HHalign sequence alignment method and three representative structure alignment methods that each use different algorithms: rigid-body alignment (TM-align), flexible alignment (FATCAT), and the residue–residue distance matrix-based method (DaliLite_v3.3 [32], a standalone version of the DALI server).

## Results

We compared CAB-align with three state-of-the-art structure alignment methods, i.e., TM-align, FATCAT, DaliLite_v3.3 (denoted as DaliLite in this study), and the HHalign sequence alignment method (except for the quality of alignment), using three evaluation approaches: quality of alignment, alignment consistency, and agreement with the SCOPe classification [33].

### Training and Benchmark Datasets

We used four datasets for training and evaluation: SISYPHUS_ID10, SCOPe_FAMILY, SCOPe_NR10, and PDB30. SISYPHUS_ID10 is a subset of the SISYPHUS dataset. SISYPHUS contains manually created structural alignments for protein pairs with nontrivial structural relationships. SISYPHUS_ID10 contains 1,627 alignments with sequence identities of <10%. SCOPe_FAMILY (2,623 domains in 903 superfamilies and 591 folds) is a subset that represents each FAMILY in the SCOPe database (Structural Classification of Proteins-Extended; Release 2.03, Oct. 2013). SCOPe_NR10 (3,542 domains in 2,160 families, 897 superfamilies, and 587 folds) is a subset with <10% shared identity from SCOPe. The lists for SCOPe_NR10 and SCOPe_FAMILY were taken from the SCOPe website (http://scop.berkeley.edu/). We then excluded low-quality structures (SPACI score [34] <0.4), NMR structures, and irregular structures, which had an atom assigned to multiple coordinates from SCOPe_FAMILY and SCOPe_NR10. In SCOPe, each protein structure is split into a domain unit and then classified according to structural and evolutionary relationships. Therefore, the impact of significant protein flexibility, such as domain arrangements, is limited in these two datasets. PDB30 is a subset of the protein data bank (PDB [35], 14 Mar 2014) with <30% shared identity. This dataset includes single-domain proteins, multidomain proteins, and all types of protein flexibility.

### Evaluation Criteria

#### AQ

First, we evaluated the alignments using the gold standard alignments in SISYPHUS_ID10 as the reference. We defined two criteria of agreement and reliability as follows:

$$\text{Agreement}=\frac{N_{c}}{L_{ref}}\;,\;\text{Reliability}=\frac{N_{c}}{L_{ali}}\;,$$ (1)

where *N*_*c*_ is the number of correctly aligned positions, *L*_*ref*_ is the length of the reference alignment, and *L*_*ali*_ is the length of the alignment under evaluation. Positions with gaps were excluded.

Second, we defined correctly aligned positions based on comparisons with the reference alignment, which reflected the evolutionary relationship. To generate the reference alignments, we used the HMM–HMM alignment program (HHalign in HH-suite 2.0 [36]), which compares two hidden Markov models (HMMs). The HMM profiles were generated using hhblits (included in HHsuit 2.0) against the Uniprot20 database (included in HHsuit 2.0). In this study, the alignments obtained from HHalign were not considered to be perfect, but they provided reliable confidence estimates for each aligned position. According to the description of HHblits, the confidence values obtained for each position by HHalign are highly correlated with the accuracy of the aligned positions. The confidence values are calculated by comparing the sequence profiles of the aligned positions. The *E*-value estimated by HHalign shares high similarity with the observed *E*-value. To assess the quality of the alignment, we defined AQ as the fraction of correctly aligned positions with estimated confidence values not less than a given threshold, as follows:

$$AQ\left(x\right)=\frac{CR_{conf\geq x}}{N_{conf\geq x}}\;,$$ (2)

where *CR*_*conf*≥*x*_ and *N*_*conf*≥*x*_ are the number of correctly aligned positions and the number of positions with confidence values not less than the given value of *x* in the reference alignment, respectively.

#### Consistency of multiple alignments

According to Sadowski and Taylor [37], we assessed the consistency among triplets of alignments, such as three alignments between *A*–*B*, *B*–*C*, and *C*–*A* for proteins *A*, *B*, and *C*. In a consistently aligned position, the following condition:

$$E\left(A_{i},B_{j}\right)\cap E\left(B_{j},C_{k}\right)\cap E\left(C_{k},A_{i}\right)\;,$$ (3)

is true, where *E*(*A*_*i*_, *B*_*j*_) denotes that position *i* in protein *A* is aligned with position *j* in protein *B*. To evaluate the consistency, we used the coverage (*Cov*) and relative rate (*Rate*) of the consistent position, which are defined as follows:

$$Cov=\frac{N_{consist}}{\text{min}\;\left\{N_{A},N_{B},N_{C}\right\}}\;,$$ (4)

$$Rate=\frac{N_{consist}}{AL_{A,B,C}}\;,$$ (5)

where *N*_*consist*_ is the number of consistent positions among the triplet alignment, *N*_*A*_ is the number of residues in protein *A*, and *AL*_*A*,*B*,*C*_ denotes the number of positions that are commonly aligned regions among the three alignments. Positions with gaps in any of the three alignments were excluded.

#### Agreement with SCOPe classification

We assessed the ability to discriminate between homologous and nonhomologous pairs of proteins based on the agreement with SCOPe classifications. We used the receiver-operating characteristic (ROC) curve and precision-recall curve (PRC) analysis. The ROC curve plots the recall against the false-positive rate (FPrate). The PRC plots the precision (or reliability) against the recall (or coverage). The precision, recall, and FPrate for a given threshold *s* are defined as follows:

$$\text{Precision}=\frac{TP_{s}}{TP_{s}+FP_{s}}\;,\;\text{Recall}=\frac{TP_{s}}{TP_{s}+FN_{s}}\;,\;\text{FPrate}=\frac{FP_{s}}{FP_{s}+TN_{s}}\;,$$ (6)

where *TP*_*s*_ is the number of true positive pairs that are correctly classified as belonging to the same class (e.g., the same family, superfamily, or fold in SCOPe) based on the threshold *s*, *FP*_*s*_ is the number of false-positive pairs that are incorrectly classified as belonging to the same class based on the threshold *s*, *TN*_*s*_ is the number of true negative pairs that are correctly assigned as belonging to a different class based on the threshold *s*, and *FN*_*s*_ is the number of false-negative pairs that are incorrectly classified as belonging to a different class based on the threshold *s*. We used the area under the ROC curve (AUC) and the area under the PRC (AUPRC) as measures of agreement with the SCOPe classification. The AUC corresponds to the probability that the proposed structural similarity score will rank a randomly selected domain pair that belongs to the same class as higher than a randomly selected pair that belongs to a different class. The AUPRC corresponds to the average precision of the proposed structural similarity score.

### Comparison of AQ

#### SISYPHUS_ID10 benchmark dataset

Table 1 shows the average agreement and reliability results for SISYPHUS_ID10 (1627 pairs) using five alignment methods. We also calculated the similarity score *S* (Eq 11) and normalized similarity score (*NormS*) (Eq 16) with the CAB-align scoring function. As shown in Table 1, DaliLite and CAB-align outperformed the other methods in terms of agreement and *N*_*c*_. In addition, these two methods generated alignments with the best and second best *S* and *NormS* on average. These results suggest that the high-quality alignments tended to have a high similarity score *S*.

**Table 1:** AQ based on SISYPHUS_ID10.

	*N*_c_ ^a^	Agreement^b^	Reliability^c^	*S* ^d^	*NormS* ^e^
**HHalign**	54.9	0.38	**0.54**	2,706.8	6.4
**CAB-align**	81.7	0.57	0.48	**4,956.8**	**11.4**
**TM-align**	74.3	0.52	0.49	4,284.0	10.0
**FATCAT**	74.1	0.52	0.47	4,240.9	9.9
**DaliLite**	**83.3**	**0.58**	0.50	4,652.0	10.8

^a^ Number of correctly aligned positions.

^b^ Agreement with the reference alignment (Eq 1).

^c^ Reliability of the evaluated alignment (Eq 1).

^d^ Similarity score obtained from the scoring function in CAB-align (Eq 11).

^e^ Normalized score obtained from the scoring function in CAB-align (Eq 16).

All data are average values per alignment.

CAB-align, contact area-based alignment; *NormS*, normalized similarity score.

**Fig 1:**
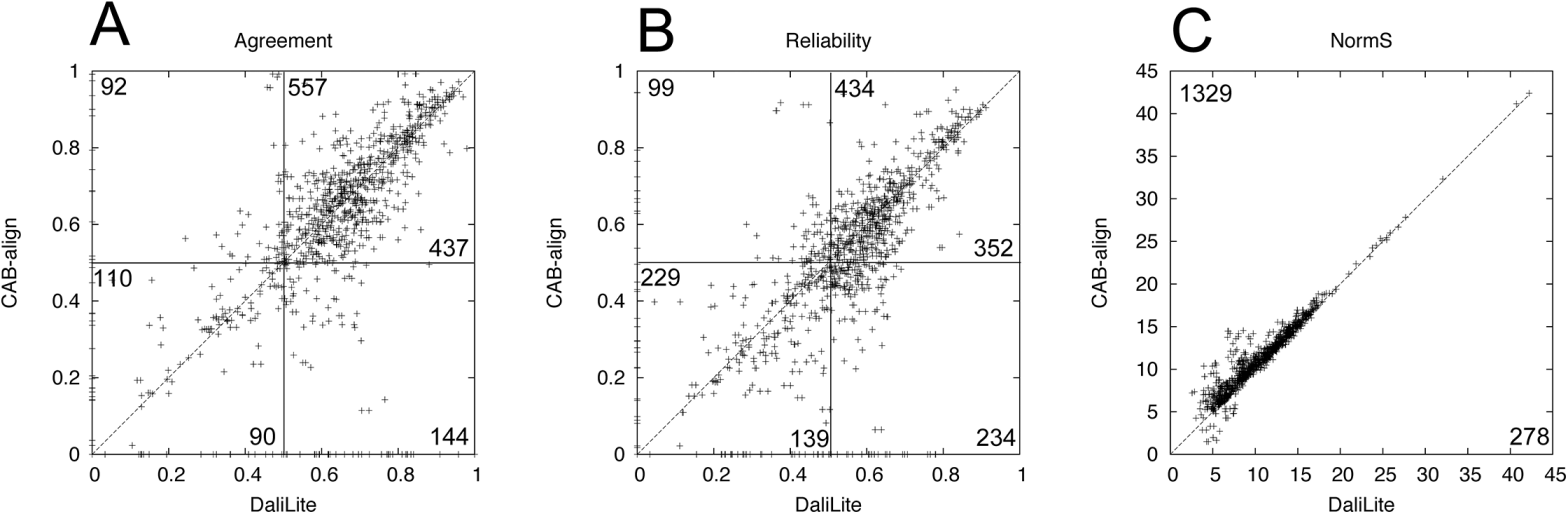
Comparison of DaliLite and CAB-align in terms of the agreement, reliability, and *NormS* values based on SISYPHUS_ID10. (**A**) Scatter plots of agreement, (**B**) reliability, and (**C**) *NormS*. The numbers of pairs belonging to each area are indicated. For example, 557 CAD-align alignments had better agreement values than DaliLite, where the agreement values for CAB-align and DaliLite were both higher than 0.5. CAB-align, contact area-based alignment.

Fig 1 compares the performance of CAB-align and DaliLite based on SISYPHUS_ID10. DaliLite performed slightly better than CAB-align in terms of the average agreement and reliability, but when we considered the total number of alignments with better performance, CAB-align (agreement: 557, reliability: 437) performed better than DaliLite (agreement: 434, reliability: 352), where both alignment methods generated high-quality alignments (agreement > 0.5, reliability > 0.5, Fig 1A and 1B). Interestingly, CAB-align and DaliLite obtained relatively similar *NormS* averages (Table 1), but Fig 1C shows that CAB-align performed better than DaliLite in terms of *NormS* for almost all pairs.

When we focused on reliability, although the *N*_*c*_ and agreement scores for HHalign were much lower than those for the other alignment methods, the reliability value was higher for HHalign than the other methods. This result suggests that the positions aligned by HHalign were relatively shorter than the other alignments generated by structural alignment methods, but the aligned regions were the most reliable.

#### SCOPe and PDB dataset

We used six benchmark datasets to compare AQ, i.e., SCOPe_NR10_all, SCOPe_NR10_e10, SCOPe_FAMILY_all, SCOPe_FAMILY_e10, PDB30_e5, and PDB30_e10. SCOPe_NR10_all and SCOPe_NR10_e10 contained 6,799 and 3,660 pairs, respectively, which were constructed from SCOPe_NR10. In the two datasets, the two proteins in each pair were chosen from the same family. Consequently, SCOPe_NR10_all and SCOPe_NR10_e10 corresponded to protein pairs where there was a confirmed evolutionary relationship at the family level in the SCOPe classification. SCOPe_FAMILY_all and SCOPe_FAMILY_e10 contained 15,790 and 5,730 protein pairs, respectively, which were constructed from the SCOPe_FAMILY. The two proteins in each pair were chosen from different families, but the same superfamily. S1 Table shows the distributions of the fold classes. PDB30_e5 and PDB30_e10 contained 182,907 and 122,626 protein pairs, respectively, which were constructed from PDB30. PDB30_e5 and PDB30_e10 were large benchmark datasets, which were constructed by focusing on flexible alignments. In SCOPe_NR10_e10, SCOPe_FAMILY_e10, and PDB30_e10, each pair also had a significant relationship with *E*-values≤10^−10^, which were estimated by HHalign. Similarly, each pair in the PDB30_e5 had an *E*-value≤10^−5^.

**Fig 2:**
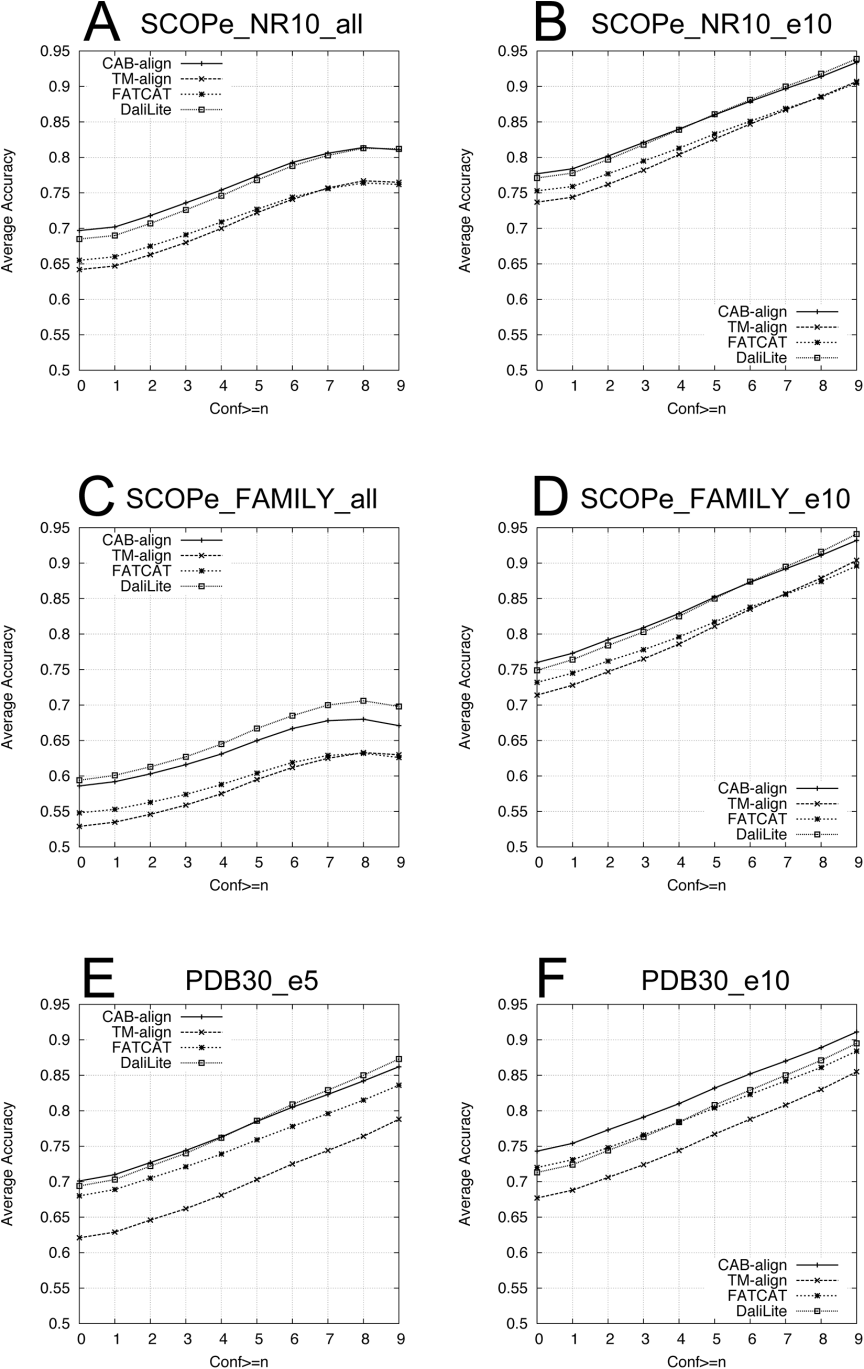
Comparison of the AQ for six benchmark datasets. (**A**) SCOPe_NR10_all (6,799 pairs), (**B**) SCOPe_NR10_e10 (3,660 pairs), (**C**) SCOPe_FAMILY_all (15,790 pairs), (**D**) SCOPe_FAMILY_e10 (5,730 pairs), (**E**) PDB30_e5 (182,907 pairs), and (**F**) PDB30_e10 (122,626 pairs). The methods are shown in order from top to bottom on the left (*n* = 0) of (**A**): CAB-align, DaliLite, FATCAT, and TM-align. CAB-align, contact area-based alignment; PDB, protein data bank.

Fig 2 shows the average values of *AQ*(*n*) (*n* = 1, 2, …, 9) for the six benchmark datasets. For all datasets (Fig 2A–2F), CAB-align and DaliLite performed better than all of the alignment methods with any confidence value obtained from HHalign. Interestingly, for five of the six benchmark datasets, CAB-align performed better than the other alignment methods in terms of *AQ*(0). As shown in Table 1, the alignments obtained from HHalign had the highest reliability. Thus, CAB-align is a robust structural alignment method that obtains high-quality alignments, which had high agreement with HHalign.

### Comparison of the Consistency of the Triplet Alignments

To assess the consistency of the alignments, we used six datasets that comprised triplets of alignments from the aforementioned protein pair datasets. In total, 7,384, 2,173, 50,630, 14,689, 1,403,291, and 790,623 triplets of alignments were obtained from SCOPe_NR10_all, SCOPe_NR10_e10, SCOPe_FAMILY_all, SCOPe_FAMILY_e10, PDB30_e5, and PDB30_e10, respectively.

**Fig 3:**
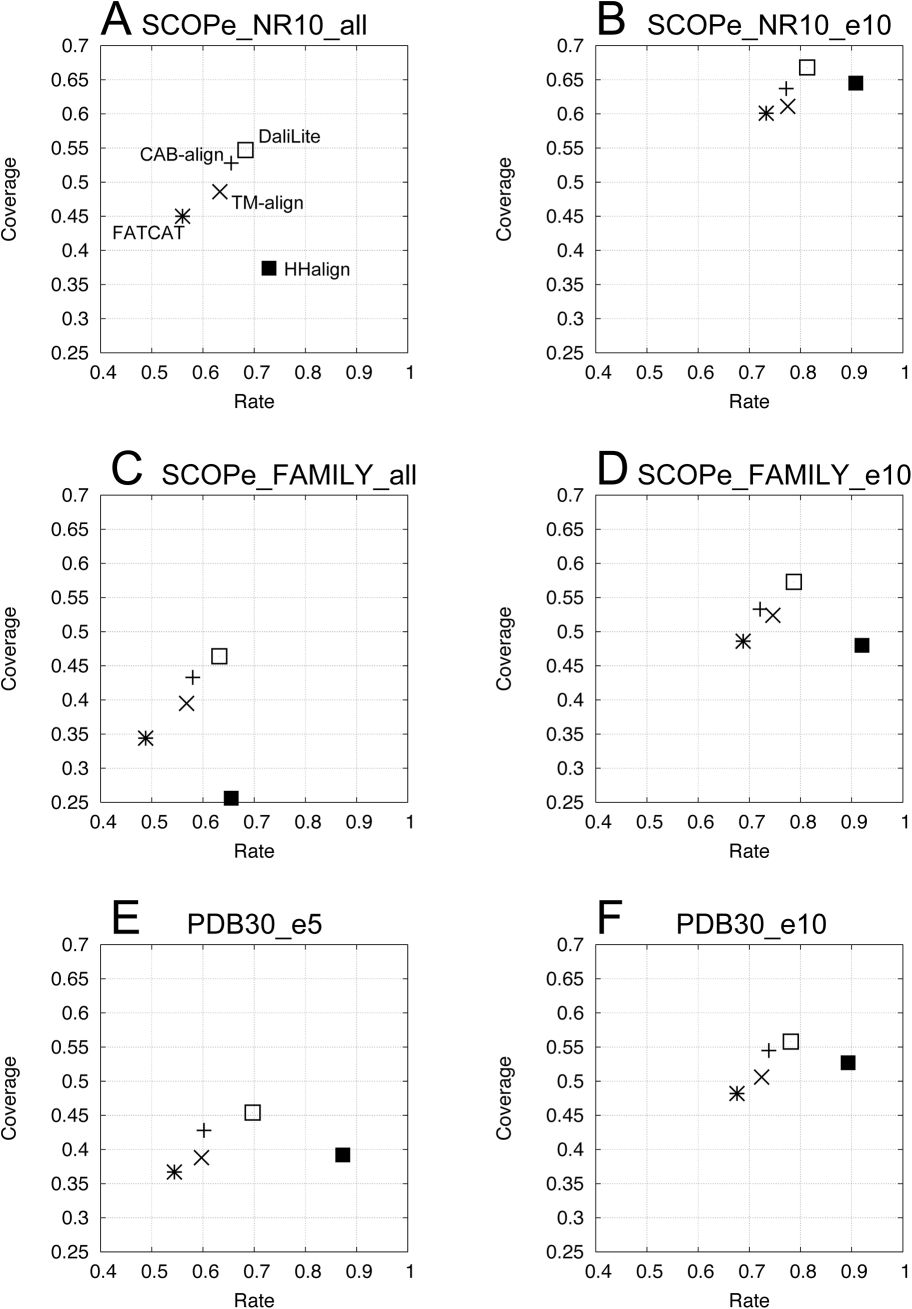
Consistency of alignments based on six datasets. (**A**) SCOPe_NR10_all (7,384 triplets), (**B**) SCOPe_NR10_e10 (2,173 triplets), (**C**) SCOPe_FAMILY_all (50,630 triplets), (**D**) SCOPe_FAMILY_e10 (14,689 triplets), (**E**) PDB30_e5 (1,403,291 triplets), and (**F**) PDB30_e10 (790,623 triplets). PDB, protein data bank.

S2 Table shows the average number of consistent positions (*N*_*consist*_), length of a commonly aligned position (*AL*), coverage (*Cov*), and relative rate (*Rate*) for the five alignment methods using the six datasets. Fig 3 shows a plot of *Cov* against *Rate*. Based on the *Rate*, HHalign performed the best with all six datasets. These results correlated with the high reliability of HHalign for the SISYPHUS_ID10 benchmark dataset (Table 1). By contrast, DaliLite had the highest *Cov* and *N*_*consist*_ scores, and CAB-align had the second highest scores for *Cov* and *N*_*consist*_, except for SCOPe_NR10_e10.

High scores are necessary for *Cov* and *Rate*, but they are not sufficient to obtain an accurate alignment. Thus, the consistency (*Cov* and *Rate*) does not directly represent the AQ. However, a high degree of consistency, which is derived from structural similarity, is necessary for improving multiple alignments. Fig 3 shows that the *Cov* score with CAB-align was comparable with that of DaliLite.

### Comparison of the Classification Performance

**Fig 4:**
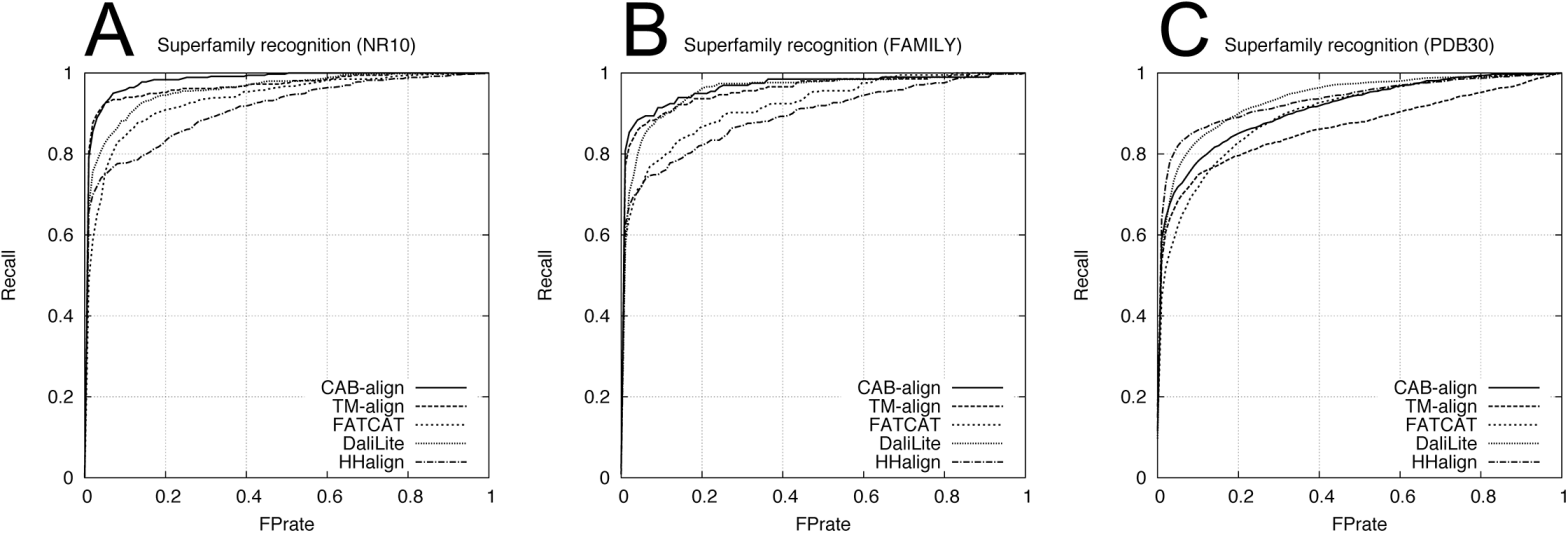
ROC curves for the five alignment methods in the superfamily recognition test. (**A**) NR10 benchmark dataset. (**B**) FAMILY benchmark dataset. (**C**) PDB30 benchmark dataset. PDB, protein data bank; ROC, receiver-operating characteristic.

**Fig 5:**
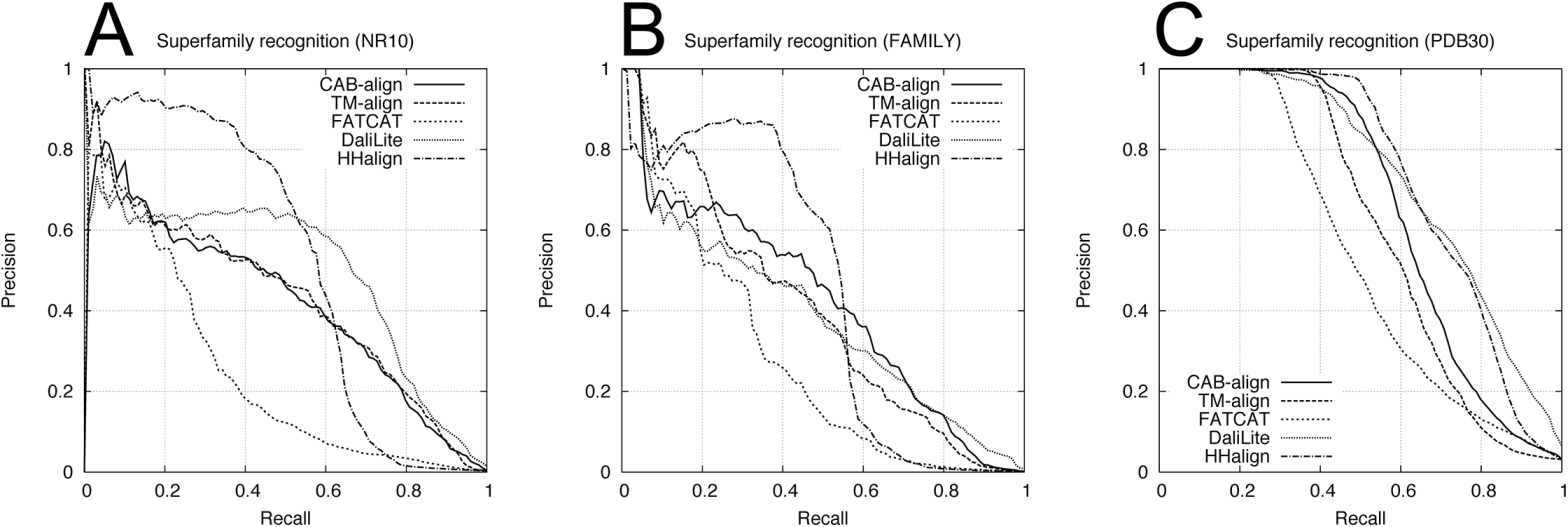
PRCs obtained using the NR10 and FAMILY datasets in the superfamily recognition test. (**A**) NR10 benchmark dataset. (**B**) FAMILY benchmark dataset. (**C**) PDB30 benchmark dataset. PDB, protein data bank; PRC, precision-recall curve.

To assess the classification performance, we used two benchmark datasets obtained from SCOPe_NR10 and SCOPe_FAMILY, which comprised single domains, where these two benchmark datasets are denoted as NR10 and FAMILY, respectively. Each benchmark dataset contained 249,500 domain pairs (500 versus 500 domains), excluding 500 same-domain pairs. The NR10 dataset contained 120 pairs from the same family, 732 pairs from the same superfamily, and 1,774 pairs with the same fold. The FAMILY dataset contained 410 pairs from the same superfamily and 1,288 pairs with the same fold. According to the SCOPe classification, the domain pairs within the same family or superfamily correspond to an evolutionary relationship. The fold only group proteins with similar structures; therefore, some domain pairs with the same fold only share structural similarity, and no evolutionary relationship has been confirmed. Furthermore, to assess the performance with multidomain proteins, we constructed a benchmark dataset containing 99,792 protein pairs selected from PDB30. In this multidomain benchmark dataset, all of the proteins contained at least two domains assigned by SCOPe. For this multidomain benchmark dataset, we defined a pair of proteins with no common domain class (i.e., family, superfamily, and fold) as negative; otherwise, the pairs were defined as positive. The multidomain benchmark dataset contained 946 pairs from the same family, 3,100 pairs from the same superfamily, and 5,762 pairs with the same fold. We evaluated the agreement between the five alignment methods and the SCOPe classifications at three levels (family, superfamily, and fold) using ROC and PRC analyses. Table 2 shows the AUC and AUPRC results for the family, superfamily, and fold recognition tests. The ROC curve and PRC for the superfamily recognition tests are plotted in Figs 4 and 5, respectively. For the two single-domain benchmark datasets (NR10 and FAMILY in Table 2), HHalign obtained outstanding AUPRC results but relatively low AUC results for the superfamily recognition tests, which corresponds to the evolutionary relationship. These results indicate that when the profile–profile alignment has sufficient sequence similarity, the homologous domain pairs can be detected with a high average precision but lower recall (or coverage) than the structure alignment methods.

**Table 2:** Classification performance with three benchmark datasets.

		Family^d^		Superfamily^e^		Fold^f^		
	Method	AUC^g^	AUPRC^h^	AUC	AUPRC	AUC	AUPRC	#^i^
**NR10** ^a^	*HHalign*	*0*.*951*	*0*.*230*	*0*.*912*	*0*.*527*	*0*.*821*	*0*.*355*	*249500*
	CAB-align	0.990	0.231	**0.984**	0.419	**0.951**	0.562	242556
	TM-align	**0.992**	**0.260**	0.970	0.428	0.945	0.549	249500
	FATCAT	0.976	0.169	0.935	0.241	0.898	0.243	249500
	DaliLite	0.975	0.171	0.958	**0.496**	0.915	**0.579**	48734
**FAMILY** ^b^	*HHalign*			*0*.*897*	*0*.*455*	*0*.*803*	*0*.*236*	*248502*
	CAB-align			**0.968**	**0.418**	0.937	0.435	236682
	TM-align			0.959	0.396	**0.952**	**0.485**	248502
	FATCAT			0.921	0.281	0.911	0.236	248502
	DaliLite			0.956	0.386	0.922	0.463	46218
**PDB30** ^c^	*HHalign*	*0*.*980*	*0*.*697*	*0*.*935*	*0*.*732*	*0*.*853*	*0*.*583*	*99792*
	CAB-align	**0.978**	0.716	0.914	0.662	0.861	0.586	91860
	TM-align	0.975	0.706	0.866	0.608	0.801	0.546	99792
	FATCAT	**0.978**	**0.717**	0.900	0.536	0.861	0.476	99792
	DaliLite	0.976	0.643	**0.937**	**0.733**	**0.892**	**0.705**	47170

^a^ Total of 249,500 domain pairs selected randomly from SCOPe_NR10.

^b^ Total of 249,500 domain pairs selected randomly from SCOPe_FAMILY.

^c^ Total of 99,792 protein pairs selected randomly from PDB30.

^d–f^ SCOPe classification.

^g^ Area under the ROC curve.

^h^ Area under the PRC.

^i^ Number of alignments calculated from the alignment program.

The best performances among the four structure alignment methods are indicated in bold.

AUC, area under the ROC curve; AUPRC, area under the PRC; CAB-align, contact area-based alignment; PDB, protein data bank; PRC, precision-recall curve; ROC, receiver-operating characteristic.

Table 2 shows the results of the comparison between CAB-align and the other structural alignment methods, which indicate that CAB-align and TM-align obtained very similar scores, with the best or second best performance in terms of AUC for the NR10 and FAMILY benchmark datasets. Interestingly, CAB-align and TM-align also obtained very similar ROC curve (Fig 4A and 4B) and PRC (Fig 5A and 5B). These results are attributable to the limitations of the structural alignment method and the use of a benchmark dataset that only contained single domains. High flexibility (such as a domain rearrangement) is rare within a single protein domain; therefore, the key advantages of CAB-align are not applicable. CAB-align only performed slightly better than TM-align in terms of the AUC in the superfamily recognition test with both NR10 and FAMILY. The PDB30 row in Table 2 shows the performance with multidomain benchmark datasets. The ROC curve and PRC are plotted in Figs 4C and 5C, respectively. DaliLite obtained the best AUC and AUPRC results in the superfamily recognition test. The performance of CAB-align was comparable with that of DaliLite. A comparison of the results obtained using the single-domain and multidomain benchmark datasets showed that CAB-align, FATCAT, and DaliLite performed better than TM-align with the multidomain benchmark dataset. These results indicate that these three alignment methods can handle potential domain–domain rearrangements because of their flexibility. In particular, the results of the single- and multidomain recognition tests indicate that CAB-align is a robust structural alignment method for predicting whether a protein pair has superfamily relationships with a high probability.

As shown in Table 2, the number of alignments returned by DaliLite was very low. To remove the bias caused by this imbalance, we constructed three additional datasets. After removing the failed alignments from NR10, FAMILY, and PDB30, we used 47,547 (NR10), 44,234 (FAMILY), and 43,219 (PDB30) alignments, which were returned by all five alignment methods. The reduced NR10 dataset contained 111 pairs from the same family, 683 pairs from the same superfamily, and 1,592 pairs with the same fold. The reduced FAMILY dataset contained 368 pairs from the same superfamily and 1,057 pairs with the same fold. The reduced PDB30 contained 888 pairs from the same family, 2,887 pairs from the same superfamily, and 5,151 pairs with the same fold.

**Fig 6:**
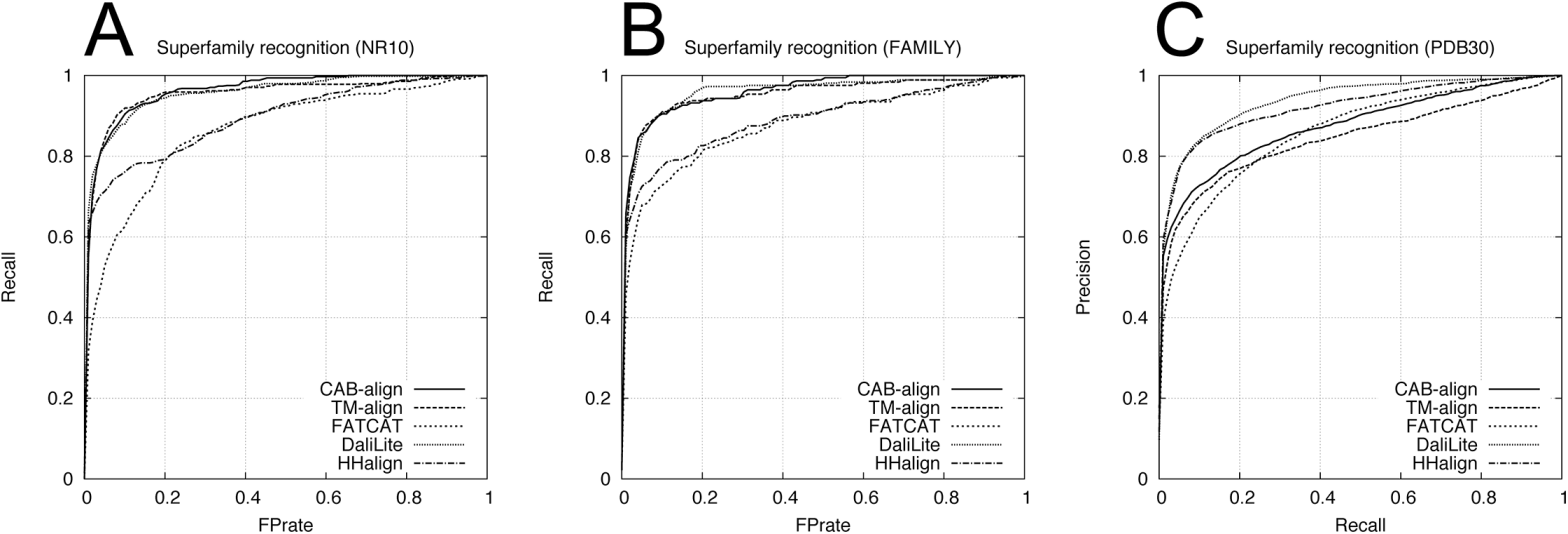
ROC curves for the five alignment methods using the alignments returned by all methods. (**A**) NR10 benchmark dataset. (**B**) FAMILY benchmark dataset. (**C**) PDB30 benchmark dataset. PDB, protein data bank; ROC, receiver-operating characteristic.

**Fig 7:**
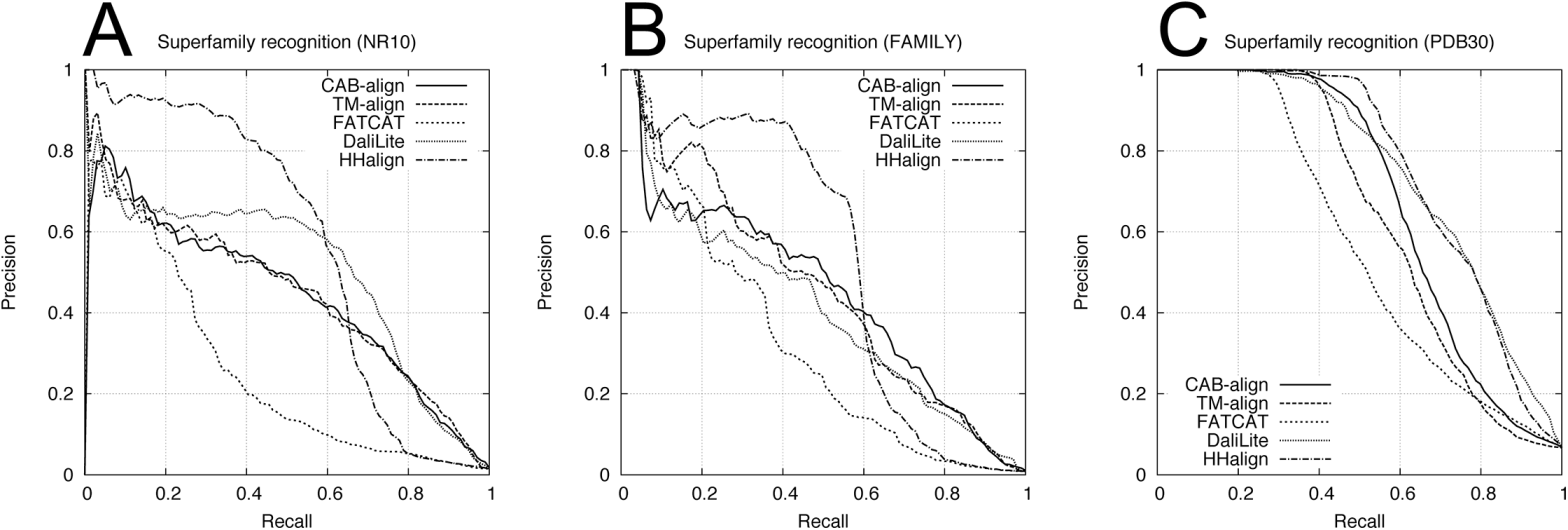
PRC for the five alignment methods using the alignments returned by all methods. (**A**) NR10 benchmark dataset. (**B**) FAMILY benchmark dataset. (**C**) PDB30 benchmark dataset. PDB, protein data bank; PRC, precision-recall curve.

Table 3 shows the AUC and AUPRC results for the family, superfamily, and fold recognition tests. The ROC and PRC obtained from the superfamily recognition tests are plotted in Figs 6 and 7, respectively. Similar to the results in Table 2, in the superfamily recognition test, CAB-align obtained the best AUC scores for the single-domain benchmark datasets and DaliLite yielded outstanding results for the multidomain benchmark dataset.

**Table 3:** Classification performance based on three benchmark datasets using the alignments returned by all methods.

		Family		Superfamily		Fold		
	Method	AUC	AUPRC	AUC	AUPRC	AUC	AUPRC	#^a^
**NR10**	*HHalign*	*0*.*927*	*0*.*240*	*0*.*896*	*0*.*572*	*0*.*795*	*0*.*437*	*47547*
	CAB-align	0.987	0.243	**0.962**	0.443	**0.924**	**0.617**	47547
	TM-align	**0.988**	**0.265**	0.954	0.447	0.919	0.597	47547
	FATCAT	0.957	0.171	0.862	0.257	0.808	0.277	47547
	DaliLite	0.975	0.173	0.957	**0.502**	0.915	0.576	47547
**FAMILY**	*HHalign*			*0*.*893*	*0*.*533*	*0*.*756*	*0*.*322*	*44234*
	CAB-align			**0.962**	0.449	0.927	0.497	44234
	TM-align			0.953	**0.457**	**0.939**	**0.544**	44234
	FATCAT			0.878	0.326	0.839	0.289	44234
	DaliLite			0.959	0.408	0.924	0.460	44234
**PDB30**	*HHalign*	*0*.*971*	*0*.*702*	*0*.*923*	*0*.*754*	*0*.*838*	*0*.*641*	*43219*
	CAB-align	0.973	**0.723**	0.877	0.680	0.815	0.625	43219
	TM-align	0.970	0.721	0.845	0.635	0.780	0.599	43219
	FATCAT	0.969	0.720	0.860	0.565	0.819	0.533	43219
	DaliLite	**0.975**	0.650	**0.939**	**0.745**	**0.894**	**0.712**	43219

^a^ Number of alignments.

Note that the results shown are based only on alignments returned by all five alignment methods.

### Contribution of Each Step

**Fig 8:**
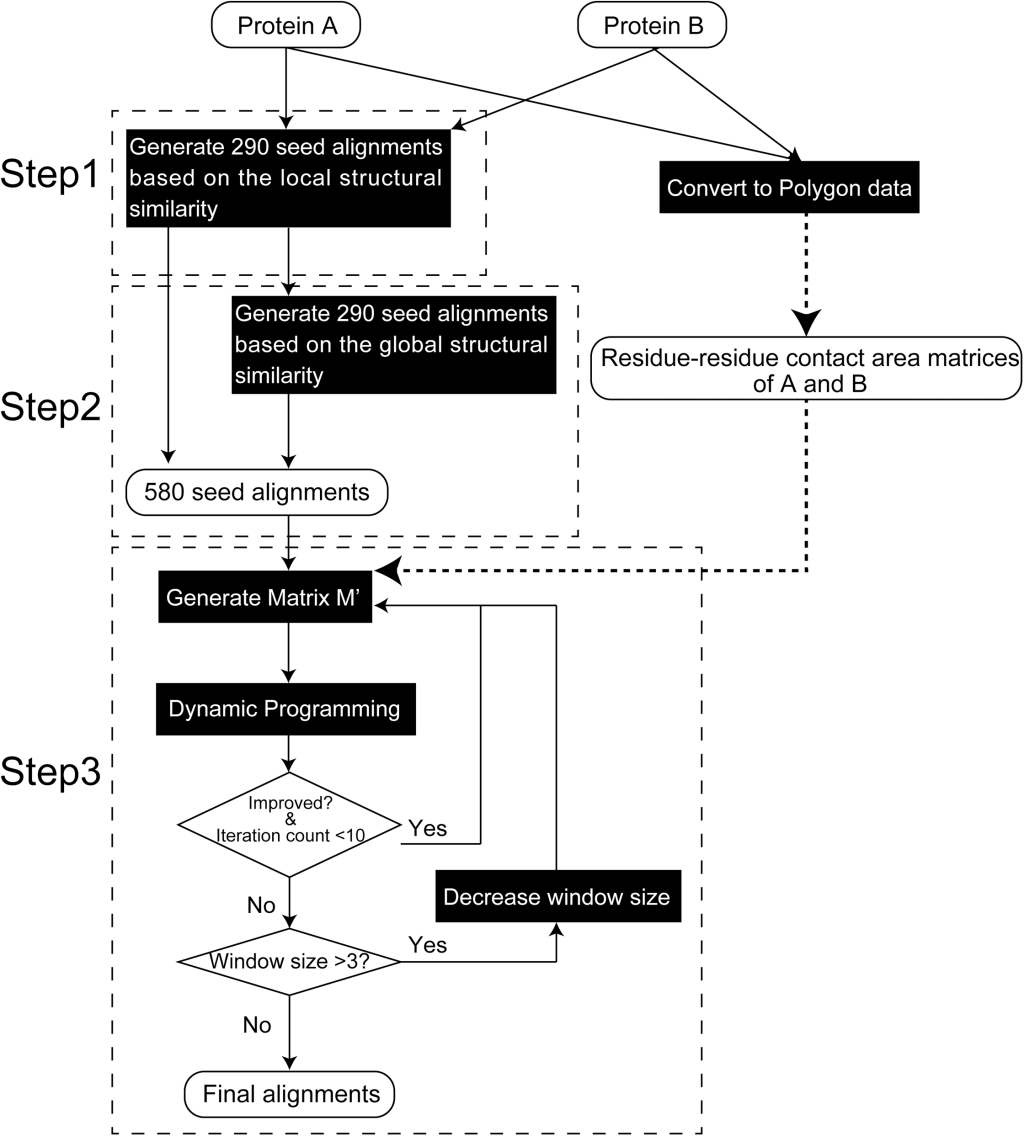
Flowchart illustrating the CAB-align procedure. CAB-align comprises three main steps. Step 1: Rigid-body alignment method based on local structural similarity. Step 2: Rigid-body alignment method based on the global 3D structure superposition. Step 3: Iterative DP based on a modified CAD-score. CAB-align, contact area-based alignment; CAD, contact area difference; DP, dynamic programming.

To assess the contribution of each step in the CAB-align method, we evaluated two components used in CAB-align denoted as step 1 and step 2. As shown in Fig 8, step 1 corresponds to the alignments obtained from the 290 initial alignments in the CAB-align procedure. These 290 initial alignments were generated by structure alignment based on the local structural similarity. Step 2 corresponds to the alignments obtained from the 580 initial alignments. These 580 initial alignments were generated by structure alignment based on the local and global structural similarities. We selected the alignments from 290 and 580 alignments using the CAB-align scoring function (Eq 11) for step 1 and step 2, respectively.

**Fig 9:**
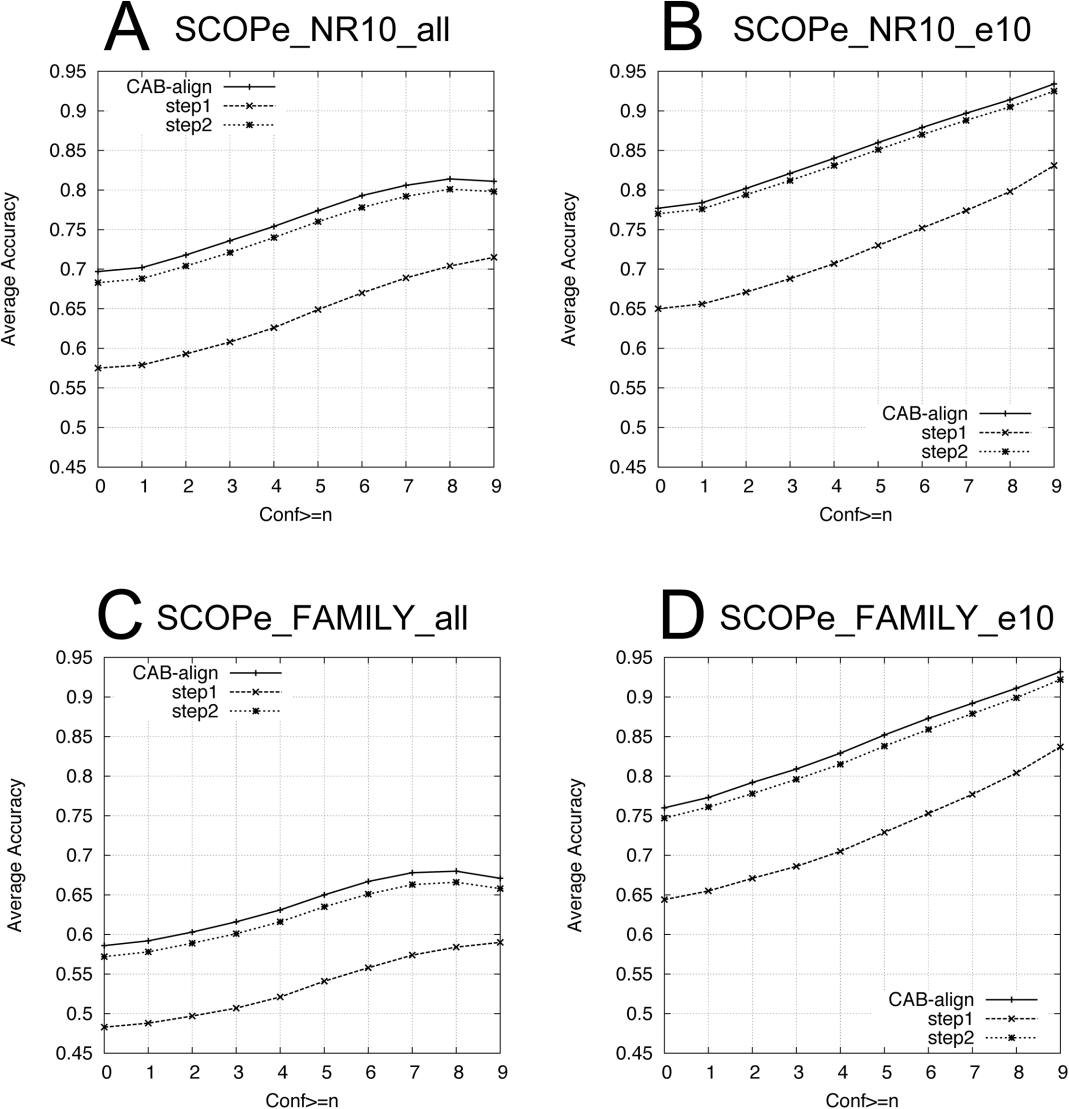
AQ of the components of CAB-align. (**A**) SCOPe_NR10_all (6,799 pairs), (**B**) SCOPe_NR10_e10 (3,660 pairs), (**C**) SCOPe_FAMILY_all (15,790 pairs), and (**D**) SCOPe_FAMILY_e10 (5,730 pairs). CAB-align, contact area-based alignment.

Table 4 shows the AQ results based on SISYPHUS_ID10. Fig 9 shows the AQ results based on SCOPe_NR10 and SCOPe_FAMILY. Table 5 shows the classification performance with the single-domain benchmark datasets.

**Table 4:** AQ of the components of CAB-align based on SISYPHUS_ID10.

	*Ncorrect*	Agreement	Reliability	*S* ^a^	*NormS* ^b^
CAB-align	81.7	0.57	0.48	**4956.8**	**11.4**
Step 1	51.9	0.36	0.31	3464.8	8.0
Step 2	**83.7**	**0.58**	**0.49**	4553.6	10.6

^a^Alignment score *S* obtained from the CAB-align scoring function (Eq 11).

^b^Normalized score *NormS* obtained from the CAB-align scoring function (Eq 16).

All of the data represent average values per alignment.

**Table 5:** AUC and AUPRC scores for the components of CAB-align.

		Family		Superfamily		Fold	
	Method	AUC	AUPRC	AUC	AUPRC	AUC	AUPRC
**NR10**	CAB-align	0.990	0.231	**0.984**	0.419	0.951	0.562
	step1	0.973	0.299	0.940	0.297	0.873	0.284
	step2	**0.992**	**0.263**	0.982	**0.468**	**0.952**	**0.564**
**FAMILY**	CAB-align			**0.968**	0.418	0.937	0.435
	Step 1			0.922	0.359	0.870	0.264
	Step 2			0.963	**0.456**	**0.949**	**0.460**

The results for CAB-align and step 2 are presented in Table 5 (SISYPHUS_ID10 benchmark dataset). For step 2, *S* and *NormS* were lower than the values derived from CAB-align. However, step2 performed marginally better than CAB-align in terms of agreement and reliability. The advantage of iterative DP in terms of the AQ was not observed with the SISPHUS_ID10 benchmark dataset. In addition, Fig 9 shows that CAB-align performed better than step 2 with all of the benchmark datasets. In terms of the classification performance, Table 5 shows that CAB-align obtained the best AUC score, but step 2 had the best AUPRC score for superfamily recognition. These results suggest that (1) step 2 dramatically improves the performance from step 1, and (2) iterative DP improves the agreement with HHalign, thereby yielding highly reliable alignments.

### Computational Time

Table 6 shows the average computation time for the five alignment methods with the SISYPHUS_ID10 benchmark dataset (1,627 pairs). We used a general Linux computing system (Intel Xeon E5506 CPU at 2.13 GHz and 12 GB memory). We found that CAB-align was about two times slower than DaliLite. As described earlier, CAB-align generates a maximum of 580 seed alignments and performs iterative DP for each alignment. Thus, the speed of CAB-align is attributable to these complex processes.

**Table 6:** Computational time.

	Seconds/pair^a^	#	Precalculation^b^	#
**HHalign**	5.97	1627	98.99	412
**CA-align**	21.98	1627	0.33	413
**TM-align**	0.79	1627		
**FATCAT**	5.38	1627		
**DaliLite**	10.45	1627		

^a^Average computational time for an alignment pair, excluding the preprocessing step.

^b^Average computational time required to preprocess a PDB file.

### Examples

**Fig 10:**
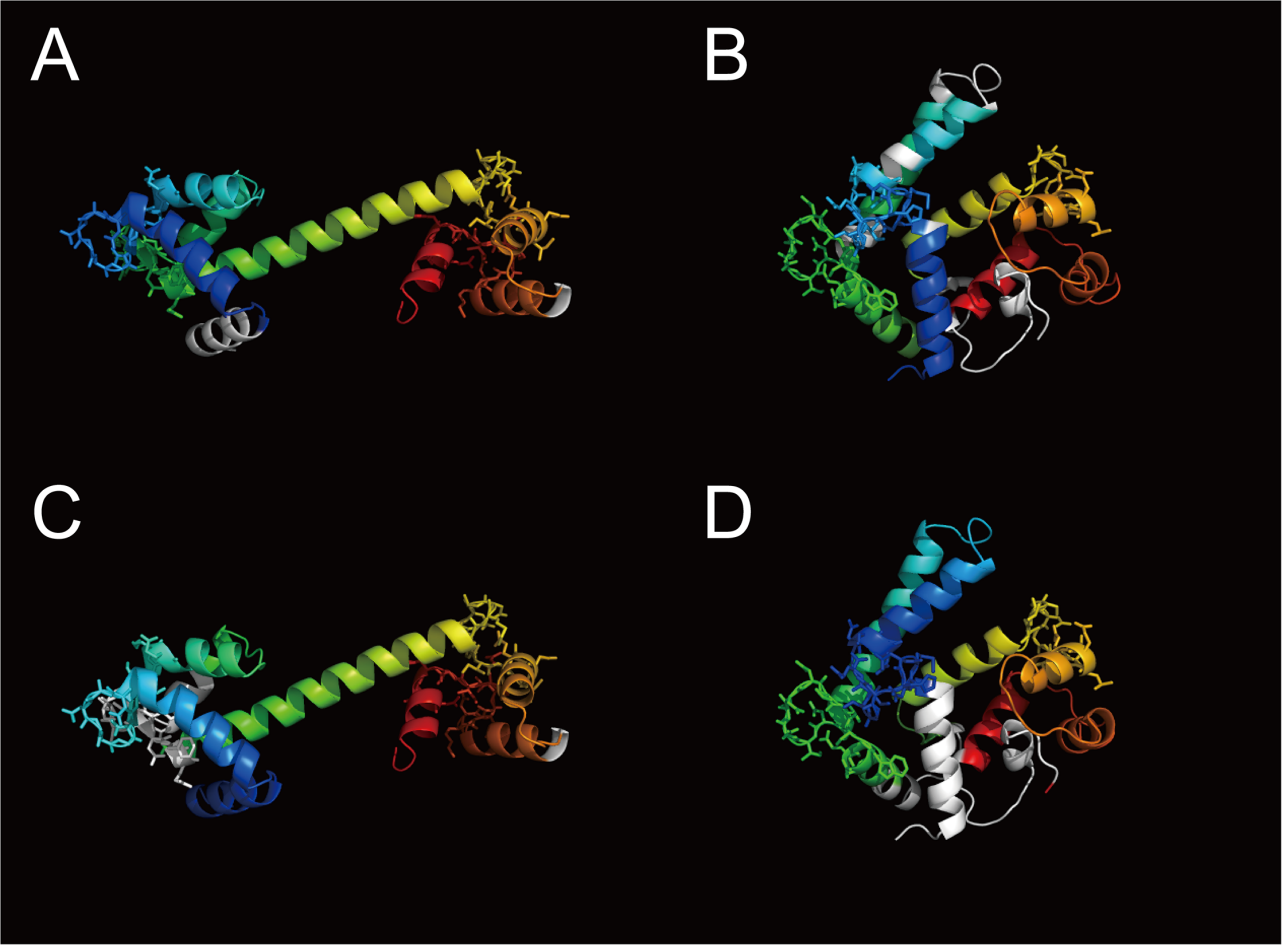
Example of a structure comparison between two calmodulin-like proteins. (**A** and **C**) Open-dumbbell conformation, 1ncx_A. (**B** and **D**) Closed conformation, 2sas_A. (**A**) Alignment of 1ncx_A by CAB-align. (**B**) Alignment of 2sas_A by CAB-align. (**C**) Alignment of 1ncx_A by DaliLite. (**D**) Alignment of 2sas_A by DaliLite. The aligned regions are rainbow color coded from blue to red. The calcium-binding regions assigned by UniProt are shown by sticks. CAB-align, contact area-based alignment.

**Fig 11:**
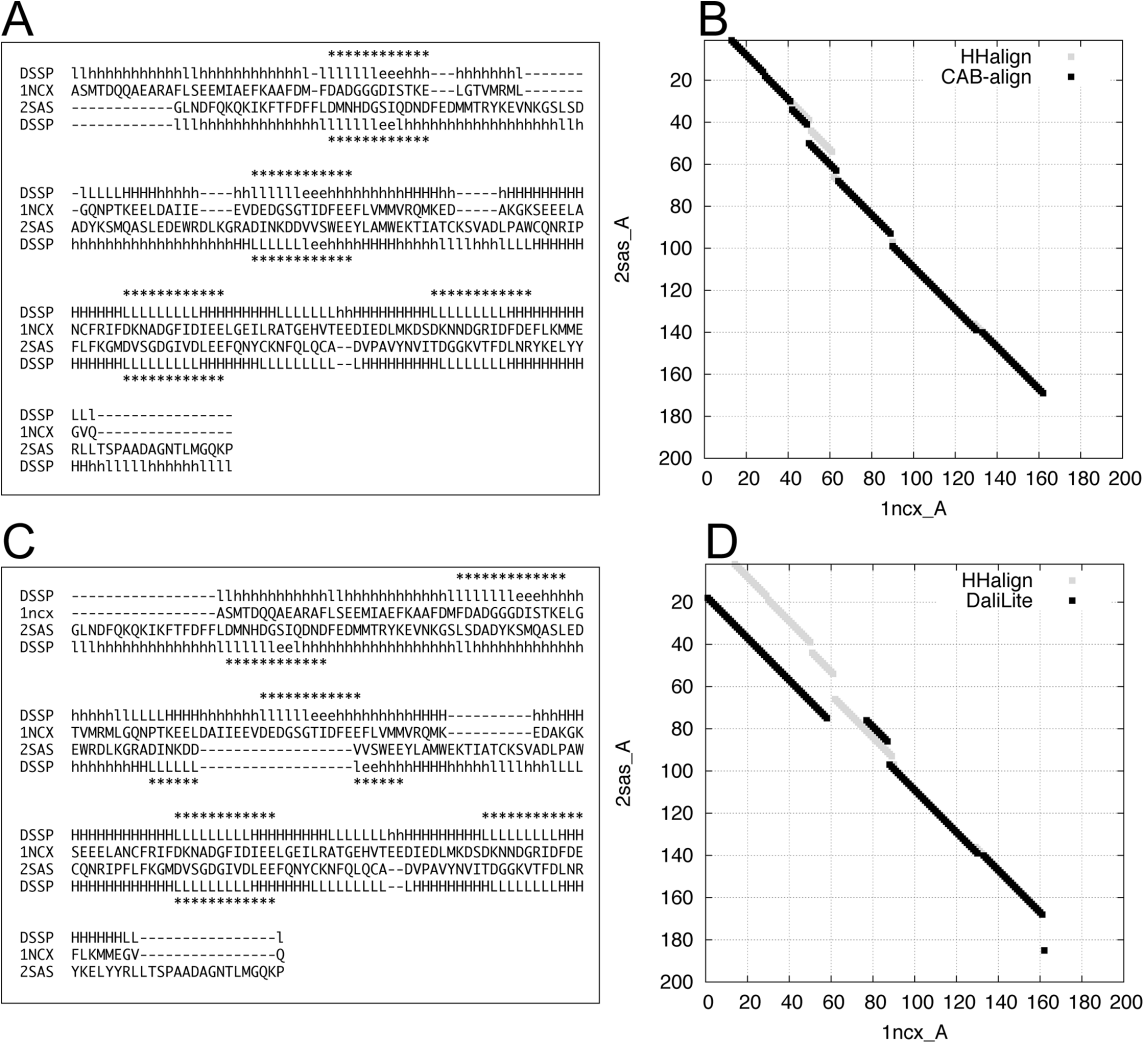
Examples of structural alignments between two calmodulin-like proteins. (**A**) Structural alignment by CAB-align. (**B**) Alignment graph produced by HHalign and CAB-align. (**C**) Structural alignment by DaliLite. (D) Alignment graph produced by HHalign and DaliLite. (**A** and **C**) The asterisks represent the calcium-binding regions. CAB-align, contact area-based alignment.

Figs 10 and 11 show examples of flexible protein comparisons between two calmodulin-like proteins (1ncx_A and 2sas_A) using CAB-align and DaliLite. Fig 10 shows the aligned regions and calcium-binding regions in the protein structures. Fig 11 shows the structural alignments, secondary structures, and calcium-binding regions assigned by UniProtKB [38]. In this case, HHalign identified a significant relationship (*E*-value = 1.2 × 10^−28^) and a reliable alignment for 146 positions between the two calmodulin-like proteins. The alignment obtained by CAB-align had a higher AQ score (*AQ*(5) = 0.80) than DaliLite (*AQ*(5) = 0.43). Moreover, Fig 11 shows that CAB-align could align three calcium-binding regions, whereas DaliLite only aligned one calcium-binding region.

**Fig 12:**
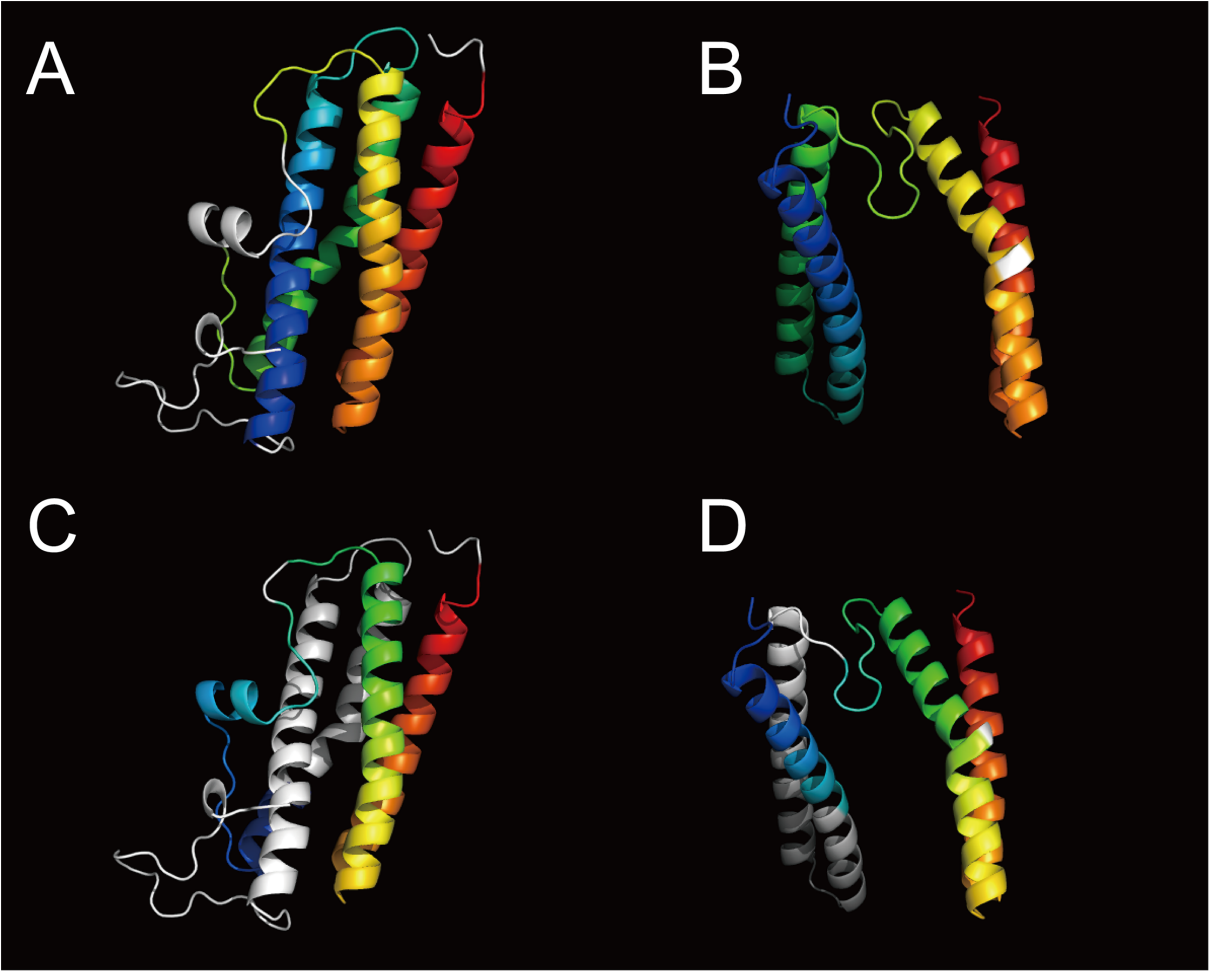
Example of a structure comparison using the SISYPHUS benchmark dataset. (**A**, **C**) 2c2f_A. (**B**, **D**) 1j30_A. (**A**) Alignment of 2c2f_A by CAB-align. (**B**) Alignment of 1j30_A by CAB-align. (**C**) Alignment of 2c2f_A by DaliLite. (**D**) Alignment of 1j30_A by DaliLite. The aligned regions are rainbow color-coded from blue to red. CAB-align, contact area-based alignment.

**Fig 13:**
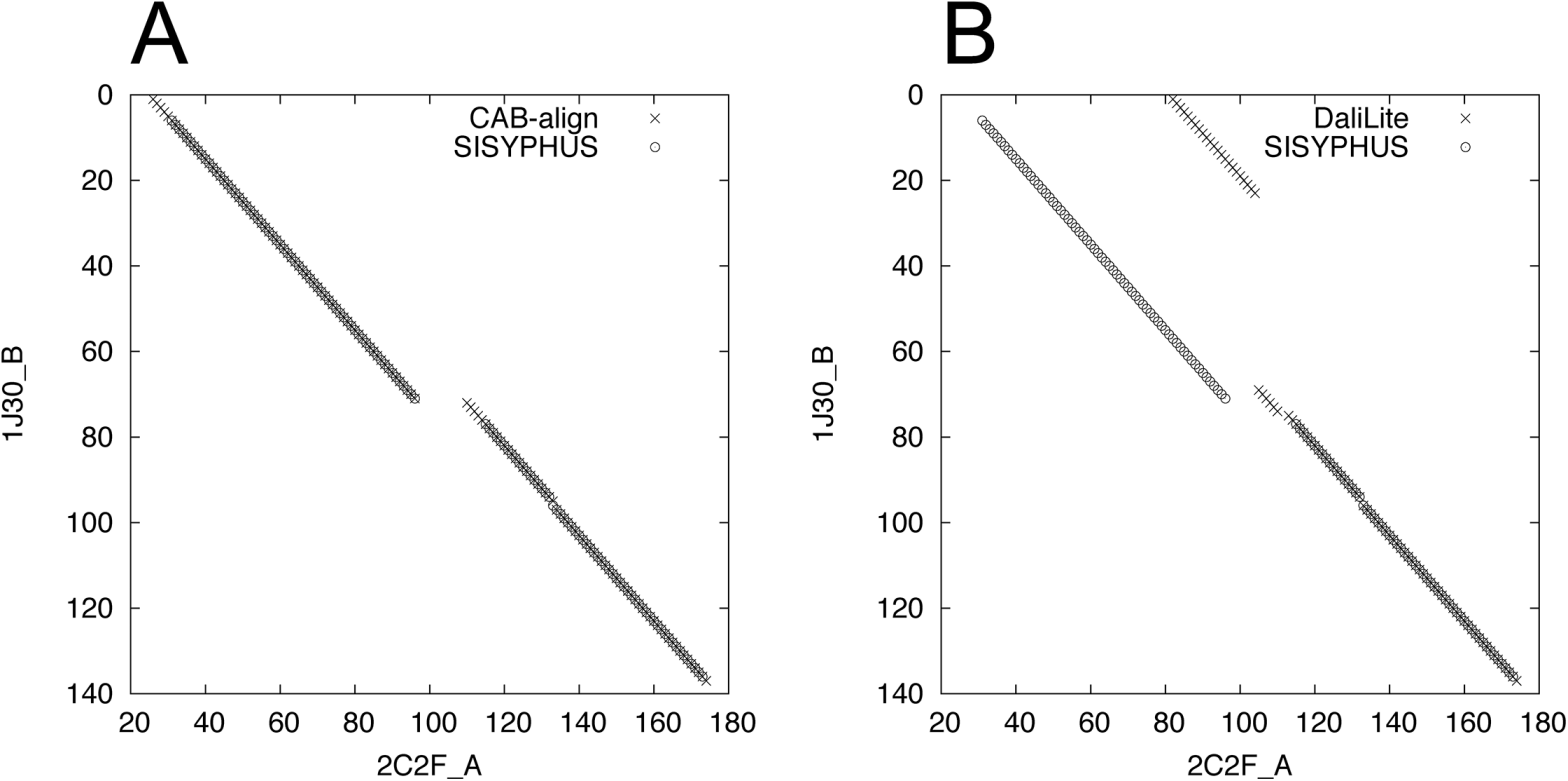
Examples of structural alignments between 2c2f_A and 1j30_A. (**A**) Alignment graph produced using SISYPHUS and CAB-align. (**B**) Alignment graph produced using SISYPHUS and DaliLite. CAB-align, contact area-based alignment.

Figs 12 and 13 show examples of protein structure comparison between 2c2f_A and 1j30_A, which are included in SISYPHUS benchmark dataset. Compared with the reference alignment in SISYPHUS (Fig 13), the alignment obtained by CAB-align had a higher agreement value (0.99) than DaliLite (0.47). CAB-align could align four helix regions for this example.

## Discussion

In this study, we applied residue–residue contact area information to protein structure alignment, and we developed a novel flexible protein structure alignment method called CAB-align. The main aim of CAB-align is to identify homologous relationships at the residue level between related protein structures whenever local or global conformational changes occur. CAB-align comprises two main steps: first, 580 initial alignments are generated based on local and global structural similarities, and second, a similarity score *S* (Eq 11) is calculated from the residue–residue contact area matrix, which is then maximized by iterative DP. To distinguish homologous pairs of proteins from nonhomologous pairs without a size dependency, the *S* value is normalized by considering the total area of the inter-residue contacts.

We evaluated the performance and advantages of CAB-align using a manually created gold standard benchmark dataset (SISYPHUS) and three large benchmark datasets, i.e., SCOPe_FAMILY, SCOPe_NR10, and PDB30. Our comparison of CAB-align with other state-of-the-art protein structure alignment methods (TM-align, FATCAT, and DaliLite) showed that CAB-align was robust, and it obtained high-quality alignments for protein pairs with known evolutionary relationships. Moreover, CAB-align generated consistent multiple alignments with high coverage and accuracy rates, which were comparable with those obtained by DaliLite. Finally, CAB-align performed well at discriminating between homologous and nonhomologous pairs of proteins in both single- and multidomain comparisons.

These results suggest future applications for the CAB-align algorithm. For example, the production of high-quality alignments will facilitate the identification of functionally important positions and the functional annotation of novel proteins. CAB-align will also allow us to discover novel evolutionary relationships at the residue level. The consistent multiple alignments obtained by CAB-align will help to identify structurally conserved regions as well as improving template-based modeling methods based on multiple templates. At present, there are many types of structural classification databases for single proteins (e.g., SCOP [39], SCOPe [33], CATH [5], PDBeFold [2], and FSSP [3,40]) or protein–protein complexes (PDBePISA [41]). The good classification performance of CAB-align is necessary for classifying new protein structures. The newly classified data will also contain previously unknown structural and evolutionary relationships.

The stand-alone software CAB-align and lists of the benchmark datasets are freely available to academic users at http://www.pharm.kitasato-u.ac.jp/bmd/bmd/Publications.html

## Materials and Methods

Fig 8 provides a flowchart that illustrates the CAB-align procedure. In this section, we describe the detailed protocols used by CAB-align.

### Surface Representation for Each Amino Acid Residue

**Fig 14:**
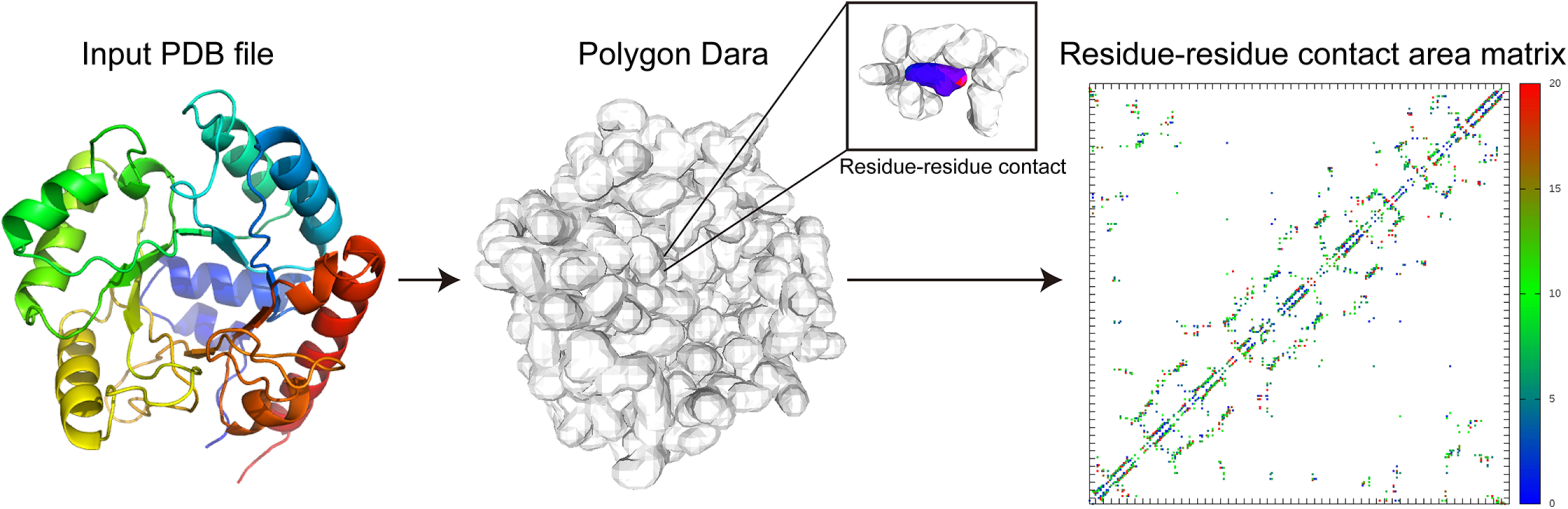
Calculation of the residue–residue contact area matrix for the input PDB file (PDBID: 1dqx_A). PDB, protein data bank.

To calculate the residue–residue contact area matrix, we used a modified smooth surface model: the simple piecewise quadratic meatball algorithm. For the surface of the *k*th amino acid residue, the shape of the surface is defined by the points *x*, which satisfy the following equations:

$$f\left(x\right)=\sum_{i}^{N_{k}}G\left(x,g_{i}\right)=1\;,$$ (7)

$$G\left(x,g_{i}\right)=\{\begin{array}{cc} 0 & if\;(cr_{i}-\left|x-g_{i}\right|\leq 0)\\ \frac{{\left(cr_{i}-\left|x-g_{i}\right|\right)}^{2}}{{\left(cr_{i}-r_{i}\right)}^{2}} & \;otherwise\;, \end{array}$$ (8)

where *N*_*k*_ is the number of atoms in the *k*th amino acid residue, *g*_*i*_ is the center of an atom *i*, *G*() is a density function, *r*_*i*_ is a van der Waals radius value of atom *i*, and *c* is a density coefficient that controls the degree of smoothness. In this study, we set *c* to 1.5, so the distances between any smoothed surfaces and the center of atoms were less than 1.5×(the van der Waals radius). We used Marching Cubes triangulation to define the triangle surface, vertices, and edges. The voxel size was set to 1.0 Å. The extracted surface was used to calculate the residue–residue contact area, where the distance between the surfaces of different residues was less than 2.8 Å. In this study, we defined an inter-residue contact area between the *l*th and *m*th residues as the sum of the surface area for the interactions between the *m*th and *l*th residues on each residue (Fig 14).

Our smooth surface model was used to approximate the solvent excluded surface (SES), which is defined by a spherical probe with a radius of 1.4 Å rolling over all the atom spheres. SES is used widely to represent the surface of a molecule, and it can be calculated by various algorithms [42,43]. Our smooth surface model correlated highly with the SES values derived from MSROLL [43]. The average deviation was 8.3 Å^2^ (6.4%), and the linear correlation coefficient was determined as 0.995 when taking 49,458 amino acid residues from a random selection of 200 protein structures.

### Generation of the Initial Alignments

To generate various initial alignments, we employed the Smith–Waterman DP algorithm [44] according to the local and global structural similarities (Steps 1 and 2 in Fig 8). The local structural similarities were generated based on the unit-vector root mean square (*URMS*) distance between all pairs of heptapeptides, as described in the MAMMOTH algorithm [9], whereas the global structural similarities were obtained using a combination of MAMMOTH and the TM-align algorithm.

First, we found 290 initial alignments based on the local structural similarity with various gap open and extension penalties. According to the MAMMOTH algorithm, the score matrix used in the DP phase is defined as follows:

$$S\left(l,m\right)=\frac{\left(URMS^{R}-URMS^{l,m}\right)}{URMS^{R}}\Delta \left(URMS^{R}-URMS^{l,m}\right),$$ (9)

$$\Delta \left(URMS^{R}-URMS^{l,m}\right)=\{\begin{array}{cc} 10, & URMS^{R}>URMS^{l,m}\\ 0, & otherwise \end{array}\;,$$ (10)

where *URMS* ^*R*^ is the expected minimum *URMS* between two random heptapeptides, which we set to 0.917. The affine gap penalty is defined as *g*(*k*) = *α* + *β*(*k* − 1), where *k* is the number of gaps, and *α* and *β* denote gap open and extension penalties, respectively. We used 11 gap open penalties ranging from 0 to 50 (step size = 5.0) and six extension penalties ranging from 0 to 10 (step size = 2.0). In total, we employed 58 combinations of *α* and *β* in the DP phase, where *α* ≥ *β*. In our study, the optimal alignments based on local similarity were not sufficient to generate the best alignment with the highest residue–residue contact area similarity. Thus, we also generated four suboptimal alignments [45] for each DP. The suboptimal alignments were generated by iteratively updating the similarity score matrix *S*(*k*, *l*). During each iteration, *S*(*k*, *l*) for the previously aligned positions was decreased by 10%, and the forward trace DP matrix was then updated and thus a new alignment was generated, which was changed slightly compared with the previously computed alignment.

We obtained more than 290 initial alignments based on the global structural similarity. These 290 alignments were then re-aligned to maximize the TM-score based on a heuristic iteration described in TM-align [10]. In this procedure, the gap open and extension penalties were set to 0.6 and 0, respectively. As result, 580 initial alignments were obtained from DP based on the local/global structural similarities.

### Iterative DP with a Contact Area Similarity Score

Following the removal of redundancies from the 580 initial alignments, the remaining alignments were subjected to iterative Smith–Waterman DP based on the residue–residue contact area matrix (Step 3 in Fig 8). To align the residue–residue contact area matrix for proteins *A* and *B*, we defined the simple similarity score as follows:

$$S=\sum_{i}^{L}\sum_{j}^{L}\theta \left(i,j\right),$$ (11)

where *i* and *j* denote a pair of aligned residues from *A* and *B* as *i* = (*i*_*A*_, *i*_*B*_) and *j* = (*j*_*A*_, *j*_*B*_), *L* is the length of the alignment, and *θ* is the similarity measure for a residue pair using the residue–residue contact area. For *θ*, we modified the CAD-score by considering two points: (1) strong overprediction of the contact is better than entirely missing the contact and (2) the similarity measure is a symmetrical function based on proteins *A* and *B*. *θ* is defined as follows:

$$\theta \left(i,j\right)=w_{i,j}\left(\text{max}\left\{0,a_{i_{A},j_{A}}^{A}-\left|a_{i_{A},j_{A}}^{A}-a_{i_{B},j_{B}}^{B}\right|\right\}+\text{max}\left\{0,a_{i_{B},j_{B}}^{B}-\left|a_{i_{A},j_{A}}^{A}-a_{i_{B},j_{B}}^{B}\right|\right\}\right),$$ (12)

$$w_{i,j}=\{\begin{array}{cc} \begin{array}{c} 0\\ 1\\ \gamma \end{array} & \begin{array}{c} \left|i_{A}-j_{A}\right|\leq 1\;or\;\left|i_{B}-j_{B}\right|\leq 1\\ \left|i_{A}-j_{A}\right|\geq 5\;and\;\left|i_{B}-j_{B}\right|\geq 5\\ otherwise \end{array} \end{array}\;,$$ (13)

where $a_{i_{A},j_{A}}^{A}$ and $a_{i_{B},j_{B}}^{B}$ are the residue–residue contact areas between aligned positions *i* and *j* of *A* and *B*, respectively, and *w*_*i*,*j*_ is a weight function used to control the overcounting of nearby contact when the sequence separation is less than five.

**Fig 15:**
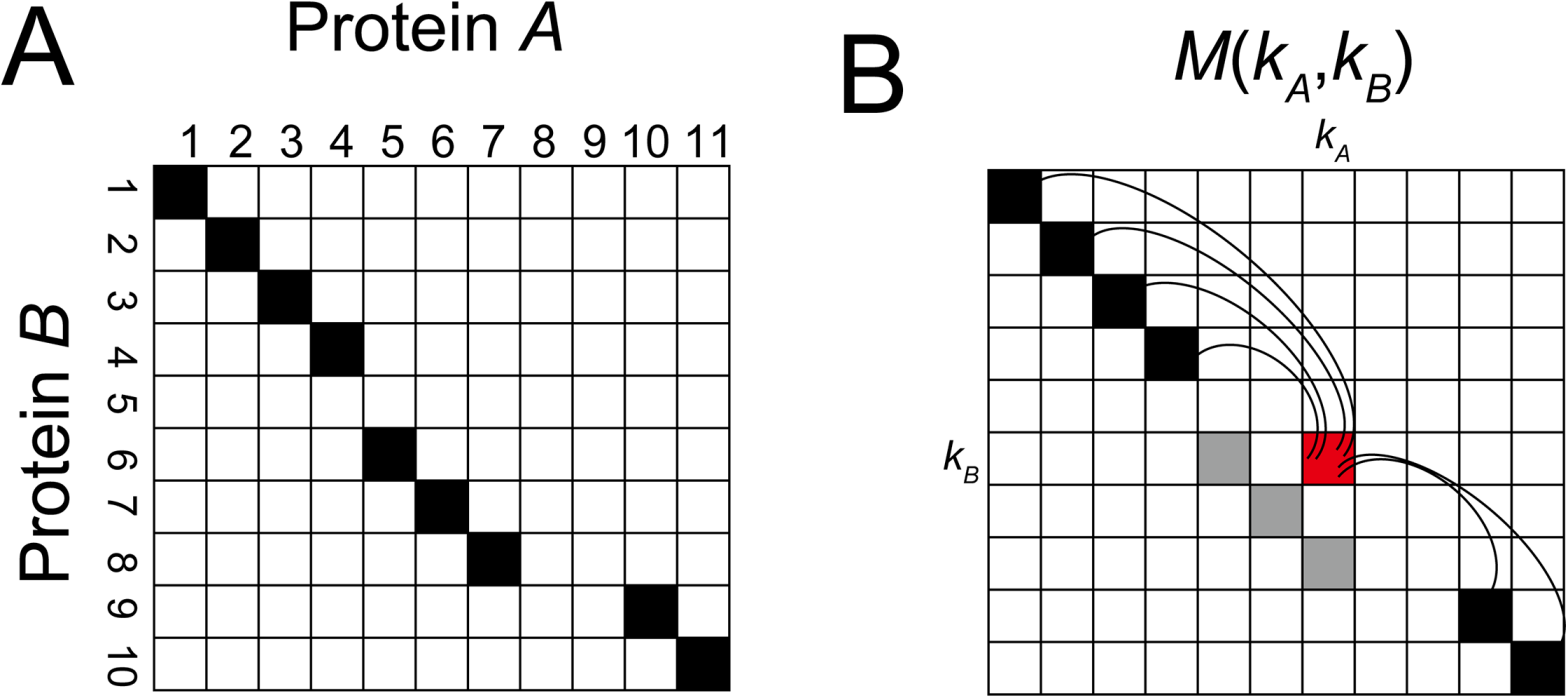
Calculation of the similarity matrix *M* from the given alignment. (**A**) The given alignment. The black cells represent the aligned pairs in protein *A* and *B*. (**B**) In the matrix *M*, the similarity score of the residue pair (*k*_*a*_,*k*_*b*_) is calculated from the comparison between other aligned positions. The gray cells are ignored. The curved lines represent the comparison between two residue pairs (Eq 12).

DP was applied to the similarity matrix to find an optimal alignment in a heuristic manner, which was calculated from the given initial alignment and the residue–residue contact area matrix. The preliminary similarity matrix *M* for all pairs of residues between proteins *A* and *B* is calculated as follows:

$$M\left(\left(k_{A},k_{B}\right)\right)=M\left(k\right)=\sum_{i,\left(k_{A}-i_{A}\right)\left(k_{B}-i_{B}\right)>0}^{L}\theta \left(i,k\right)\;,$$ (14)

where *M*(*k*) denotes the similarity between residues *k*_*A*_ in protein *A* and *k*_*B*_ in protein *B* when the other aligned positions *i* = (*i*_*A*_, *i*_*B*_) are not changed for the given alignment (Fig 15). As shown in Fig 15B and Eq 14, the conflicted pairs are ignored when (*k*_*A*_ − *i*_*A*_)(*k*_*B*_ − *i*_*B*_) ≤ 0.

**Fig 16:**
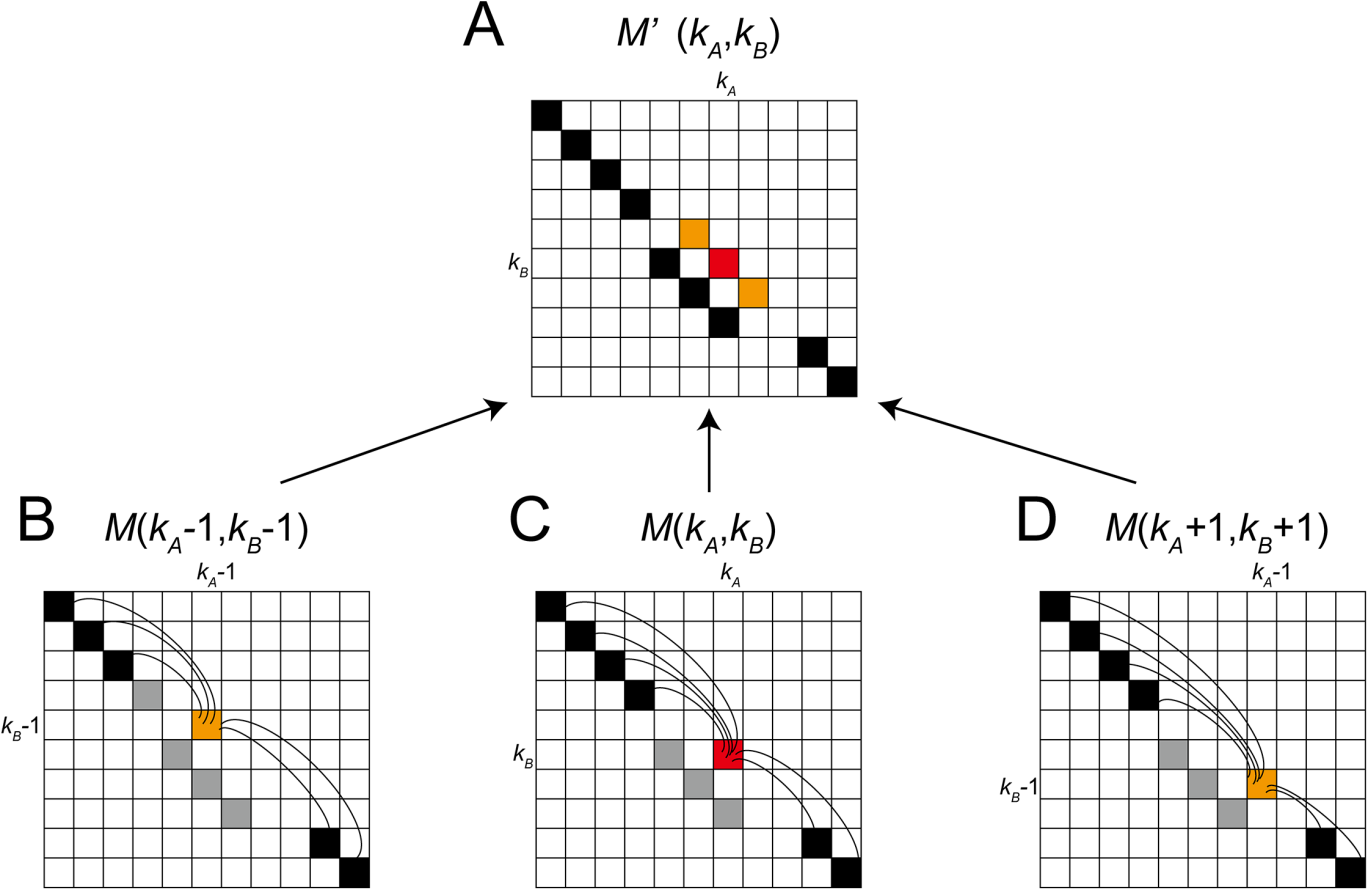
Calculation of the similarity matrix *M*′ with a window size of three. (**A**) In the matrix *M*′, the similarity score for the residue pair (*k*_*a*_,*k*_*b*_) is calculated from the two orange cells (*M*(*k*_*a*_–1,*k*_*b*_–1) and *M*(*k*_*a*_+1,*k*_*b*_+1)) and the red cell *M*(*k*_*a*_,*k*_*b*_). (**B**) Similarity score for the residue pair (*k*_*a*_–1,*k*_*b*_–1). (**C**) Similarity score for the residue pair (*k*_*a*_,*k*_*b*_). (**D**) Similarity score for the residue pair (*k*_*a*_+1,*k*_*b*_+1). The black cells represent the aligned positions in the given alignment. The gray cells represent the ignored pairs.

However, *M* is too restrictive to improve the alignment by DP and only the local optimal alignments were obtained in our study. Therefore, *M* is converted into *M*' by considering neighboring residues using a sliding window. *M*' is defined as follows:

$$M'\left(\left(k_{A},k_{B}\right)\right)=M'\left(k\right)=\frac{1}{2N_{w}+1}\sum_{\begin{array}{l} p=1,\\ 0<k_{A}-N_{w}+p\leq N_{A},\\ 0<k_{B}-N_{w}+p\leq N_{B} \end{array}}^{2N_{w}+1}M\left(\left(k_{A}-N_{w}+p,k_{B}-N_{w}+p\right)\right)\;,$$ (15)

where *N*_*A*_ and *N*_*B*_ are the number of residues in protein *A* and *B*, respectively, and *N*_*w*_ defines the size of the window. Thus, 2*N*_*w*_ + 1 corresponds to the window size. *M*' approximates the similarity between the two fragments, i.e., *N*_*w*_ residues around *k*_*A*_ and *k*_*B*_ (Fig 16).

**Fig 17:**
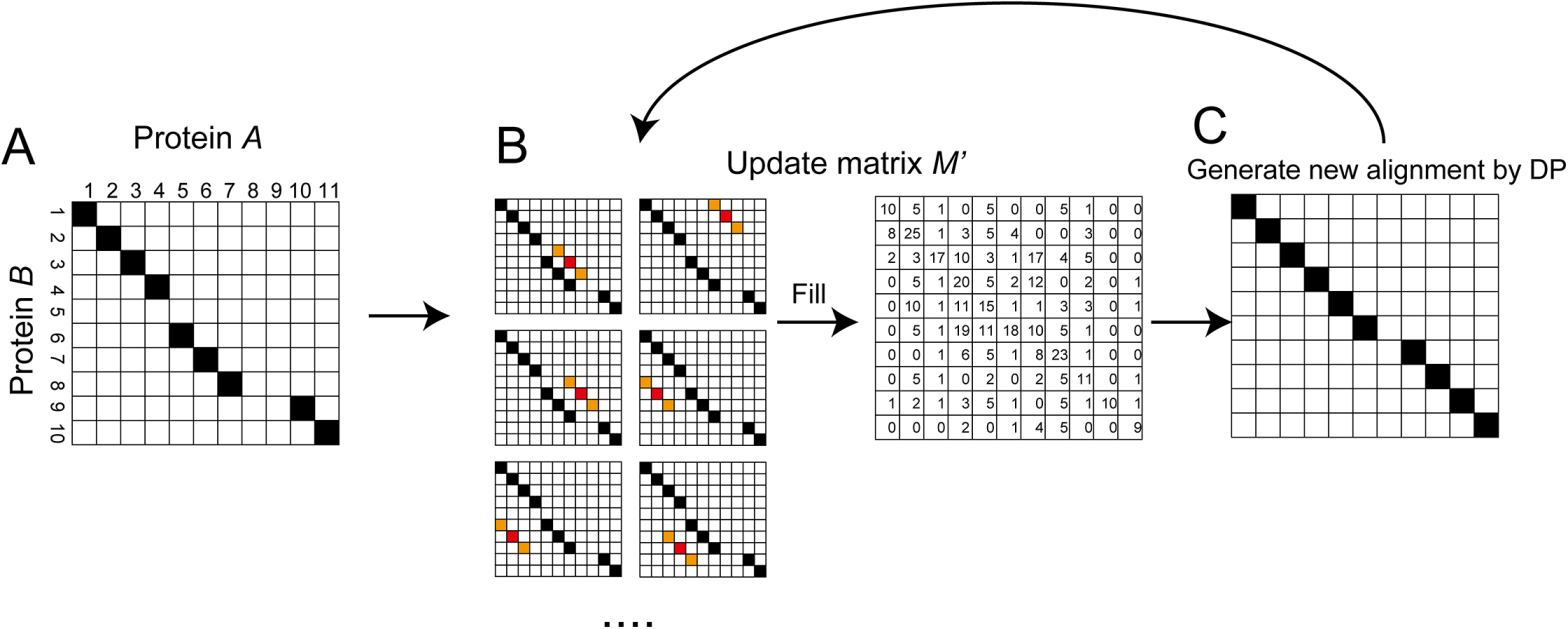
Protocols employed for iterative DP with a window size of three. (**A**) The given alignment. (**B**) The similarity score matrix *M*′ is updated by the alignment. (**C**) DP is performed using the *M*′ and a new alignment is generated. DP, dynamic programming.

Starting from the given initial alignment, the heuristic method (iterative DP) was applied to obtain an optimal alignment. Step 3 in Figs 8 and 17 summarizes the iterative DP. First, the window size was set to 11 (i.e.,., *N*_*w*_ = 5), and *M*' was then calculated from the given alignment (Fig 17A and 17B). DP was performed and after each round of DP, the similarity matrix *M*' was updated based on the alignment obtained (Fig 17B). The iterative DP was repeated until the similarity score *S* converged, or 10 iterations were reached. After this set of iterations, the size of the window was reduced, and iterative DP was then repeated again. The size of the window was reduced gradually for *N*_*w*_ = {5, 3, 1} (Fig 8). Finally, the best alignment was obtained with the highest *S* value. The parameters *γ* (Eq 13) and the gap open penalty in iterative DP were optimized based on a training dataset (a subset of the SCOPe_FAMILY) by maximizing the average *AQ*(5). The training dataset contained 4,144 protein pairs with a significant evolutionary relationship (*E*-value ≤10^−10^) and a reliable alignment length (≥100). Moreover, to confirm the evolutionary relationship, both proteins in the pair had to belong to the same superfamily. Our parameter optimization indicated that the optimal parameter was *γ* = 0.5, and the gap open penalty in iterative DP was 90.

### Normalized Similarity Score

**Fig 18:**
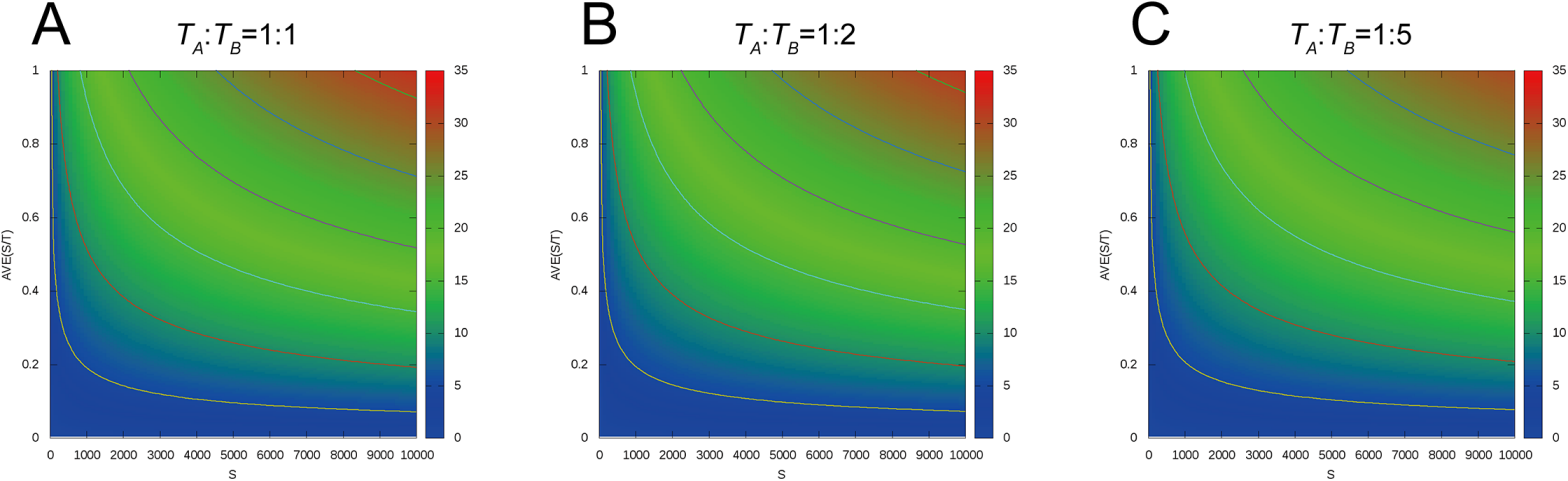
Distributions of the normalized score, *NormS*. The contour lines are plotted at interval values of 10.0 for *NormS*. The vertical line represents the average rate of *S* (i.e., 0.5*(*S*/*T*_*A*_+*S*/*T*_*B*_)). The horizontal line represents the similarity score *S*. (**A**) *T*_*A*_:*T*_*B*_ = 1:1, (**B**) *T*_*A*_:*T*_*B*_ = 1:2, and (**C**) *T*_*A*_:*T*_*B*_ = 1:5. *NormS*, normalized similarity score.

The raw similarity score *S* represents the degree of a common inter-residue contact area between two proteins according to the alignment, but it depends on the size of the proteins. A pair of large proteins usually has a large *S* value. Due to this size dependency, *S* cannot be used to compare the similarities of different protein pairs. The main aim of the normalization is to distinguish homologous pairs of proteins from nonhomologous pairs. Thus, we propose a *NormS* that considers the total inter-residue contact area as follows:

$$NormS=\frac{S}{{\left(T_{A}\right)}^{pow}}+\frac{S}{{\left(T_{B}\right)}^{pow}}\;,$$ (16)

where *S* is the similarity score of the alignment (Eq 11), *T*_*A*_ and *T*_*B*_ are the total inter-residue contact areas in proteins *A* and *B*, respectively, and *pow* is a multiplier factor. When *pow* is set to 0.7, the *NormS* has two interesting features: (1) the protein pair with a higher *S* tends to have a higher *NormS*, and (2) the higher relative rate of *S* against *T*_*A*_ and *T*_*B*_ tends to yield a higher *NormS* (Fig 18). The parameter *pow* was optimized based on 500 proteins from the SCOPe_FAMILY by maximizing the AUC for superfamily recognition. Based on the parameter optimization for *pow*, a value of 0.7 was found to be optimal.

## Supporting Information

S1 Table Distributions of the SCOPe classes. (DOCX)

S2 Table Consistency of triplet alignments based on the six datasets. (DOCX)
