# Supplementary material for: CAB-Align: A Flexible Protein Structure Alignment Method Based on the Residue-Residue Contact Area
Source: PLoS One. 2015 Oct 26;10(10):e0141440. doi: 10.1371/journal.pone.0141440 (PMC4621035; doi:10.1371/journal.pone.0141440)
Supplement: S1 Table — (DOCX) [file pone.0141440.s001.docx]

**S1 Table. Distributions of the SCOPe class.**

| **Fold class** | **SCOPe_NR10_all** | **SCOPe_NR10_e10** | **SCOPe_FAMILY_all** | **SCOPe_FAMILY_e10** |
| --- | --- | --- | --- | --- |
| **all alpha proteins** | 951 | 524 | 2,843 | 564 |
| **all beta proteins** | 2,051 | 811 | 2,952 | 383 |
| **alpha/beta proteins** | 2,509 | 1,552 | 6,559 | 3,679 |
| **alpha+beta proteins** | 1,115 | 706 | 3,214 | 1,035 |
| **multi-domain proteins** | 29 | 25 | 70 | 30 |
| **membrane and cell surface proteins and peptides** | 16 | 2 | 20 | 10 |
| **small proteins** | 128 | 40 | 132 | 29 |
| **Total** | 6,799 | 3,660 | 15,790 | 5,730 |
