# Supplementary material for: CAB-Align: A Flexible Protein Structure Alignment Method Based on the Residue-Residue Contact Area
Source: PLoS One. 2015 Oct 26;10(10):e0141440. doi: 10.1371/journal.pone.0141440 (PMC4621035; doi:10.1371/journal.pone.0141440)
Supplement: S2 Table — (DOCX) [file pone.0141440.s002.docx]

**S2 Table. Consistency of triplet alignments on the six datasets.**

|  | *N_consist_* | *AL* | *Rate* | *Cov* |
| --- | --- | --- | --- | --- |
| SCOPe_NR10_all (7,384) |  |  |  |  |
| HHalign | 49.6 | 57.1 | 0.73 | 0.37 |
| CAB-align | 67.1 | **100.6** | 0.66 | 0.53 |
| TM-align | 62.7 | 94.9 | 0.63 | 0.49 |
| FATCAT | 56.8 | 95.6 | 0.56 | 0.45 |
| DaliLite | **70.3** | 97.3 | 0.68 | 0.55 |
| SCOPe_NR10_e10 (2,173) |  |  |  |  |
| HHalign | 99.2 | 109.6 | **0.91** | 0.65 |
| CAB-align | 99.2 | **129.9** | 0.77 | 0.64 |
| TM-align | 94.9 | 124.1 | 0.78 | 0.61 |
| FATCAT | 91.9 | 127.1 | 0.73 | 0.60 |
| DaliLite | **104.2** | 128.3 | 0.81 | **0.67** |
| SCOPe_FAMILY_all (50,630) |  |  |  |  |
| HHalign | 41.8 | 47.6 | **0.66** | 0.26 |
| CAB-align | 65.7 | **109.0** | 0.58 | 0.43 |
| TM-align | 61.1 | 98.7 | 0.57 | 0.40 |
| FATCAT | 52.9 | 98.7 | 0.49 | 0.34 |
| DaliLite | **70.8** | 100.2 | 0.63 | **0.46** |
| SCOPe_FAMILY_e10 (14,689) |  |  |  |  |
| HHalign | 95.8 | 105.0 | **0.92** | 0.48 |
| CAB-align | 106.9 | **149.5** | 0.72 | 0.53 |
| TM-align | 105.6 | 141.7 | 0.75 | 0.52 |
| FATCAT | 97.2 | 141.7 | 0.69 | 0.49 |
| DaliLite | **115.0** | 146.5 | 0.79 | **0.57** |
| PDB30_e5 (1,403,291) |  |  |  |  |
| HHalign | 84.7 | 96.5 | **0.87** | 0.39 |
| CAB-align | 93.8 | **156.6** | 0.60 | 0.43 |
| TM-align | 85.1 | 132.5 | 0.60 | 0.39 |
| FATCAT | 79.4 | 142.7 | 0.54 | 0.37 |
| DaliLite | **101.3** | 139.8 | 0.70 | **0.45** |
| PDB30_e10 (790,623) |  |  |  |  |
| HHalign | 114.3 | 128.6 | **0.89** | 0.53 |
| CAB-align | 119.7 | **164.5** | 0.74 | 0.55 |
| TM-align | 110.9 | 154.2 | 0.72 | 0.51 |
| FATCAT | 104.7 | 157.1 | 0.68 | 0.48 |
| DaliLite | **123.2** | 158.1 | 0.78 | **0.56** |

All data are average values per a triplet alignment
